# Canonical Causal Diagrams to Guide the Treatment of Missing Data in Epidemiologic Studies

**DOI:** 10.1093/aje/kwy173

**Published:** 2018-08-14

**Authors:** Margarita Moreno-Betancur, Katherine J Lee, Finbarr P Leacy, Ian R White, Julie A Simpson, John B Carlin

**Affiliations:** 1 Clinical Epidemiology and Biostatistics Unit, Murdoch Children’s Research Institute, Melbourne, Victoria, Australia; 2 Centre for Epidemiology and Biostatistics, Melbourne School of Population and Global Health, University of Melbourne, Melbourne, Victoria, Australia; 3 Department of Paediatrics, Melbourne Medical School, University of Melbourne, Melbourne, Victoria, Australia; 4 Data Science Centre, Royal College of Surgeons in Ireland, Dublin, Ireland; 5 MRC Clinical Trials Unit, London, United Kingdom

**Keywords:** directed acyclic graphs, missing data, missing at random assumption, missing not at random assumption, multiple imputation, potential outcomes, recoverability, sensitivity analysis

## Abstract

With incomplete data, the “missing at random” (MAR) assumption is widely understood to enable unbiased estimation with appropriate methods. While the need to assess the plausibility of MAR and to perform sensitivity analyses considering “missing not at random” (MNAR) scenarios has been emphasized, the practical difficulty of these tasks is rarely acknowledged. With multivariable missingness, what MAR means is difficult to grasp, and in many MNAR scenarios unbiased estimation is possible using methods commonly associated with MAR. Directed acyclic graphs (DAGs) have been proposed as an alternative framework for specifying practically accessible assumptions beyond the MAR-MNAR dichotomy. However, there is currently no general algorithm for deciding how to handle the missing data given a specific DAG. Here we construct “canonical” DAGs capturing typical missingness mechanisms in epidemiologic studies with incomplete data on exposure, outcome, and confounding factors. For each DAG, we determine whether common target parameters are “recoverable,” meaning that they can be expressed as functions of the available data distribution and thus estimated consistently, or whether sensitivity analyses are necessary. We investigate the performance of available-case and multiple-imputation procedures. Using data from waves 1–3 of the Longitudinal Study of Australian Children (2004–2008), we illustrate how our findings can guide the treatment of missing data in point-exposure studies.

Epidemiologic studies often suffer from missing data on multiple variables, including the exposure of interest, the outcome, and confounding factors. Methods such as multiple imputation (1, 2) allow unbiased estimation of all possible target parameters (e.g., mean values, regression-adjusted associations) if the “missing at random” (MAR) assumption holds. Investigators are thus urged to assess the plausibility of this assumption and encouraged to perform structured sensitivity analyses to examine the robustness of their conclusions to departures from MAR (3). Such analyses usually entail the elicitation of sensitivity parameters from subject-matter experts, requiring considerable effort.

However, there is a shortage of guidance on how investigators should assess the plausibility of MAR in practice. Seaman et al. (4) highlighted a lack of clarity about the definition of MAR in much of the missing-data literature, and they showed that a precisely defined condition called “everywhere MAR” (which is what we mean by MAR hereafter) is needed to guarantee valid frequentist inferences based on the likelihood—including methods such as multiple imputation. As Mealli and Rubin (5) noted, MAR is not an assumption about conditional independencies between variables, as is often thought, but about the nondependence of a function on one of its arguments. This is difficult to assess on the basis of substantive knowledge (6), which is crucial in the multivariable missingness setting (7–9). In particular, the stringency of MAR in general problems with multivariable missingness is poorly understood (4–6, 10, 11). Meanwhile, although MAR is sufficient for unbiased estimation, it is not necessary: In problems with multivariable missingness, many “missing not at random” (MNAR) scenarios allow unbiased estimation of all or some parameters using standard implementations of multiple imputation or even a complete-case analysis. This has been noted in specific situations (7, 12), but there is little clarity as to how researchers can distinguish such MNAR settings from those in which sensitivity analyses are required.

Mohan and various colleagues (9, 13–15) proposed causal directed acyclic graphs (DAGs) as intuitive tools for depicting practically accessible and finer-grained missingness assumptions, beyond the MAR-MNAR dichotomy. This is a promising framework to guide the treatment of missing data. However, its practical applicability is currently impeded by the lack of a general algorithm with which to ascertain, given an arbitrary DAG, the nonparametric identifiability or “recoverability” (9, 13–15) of target parameters. That is, there is no general algorithm for determining from a DAG whether a given parameter can be estimated consistently using available data (and, if so, how) or whether sensitivity analyses are needed (16).

In this paper, we construct “canonical” DAGs capturing typical missingness mechanisms in the point-exposure study design with incomplete data on the exposure, outcome, and confounders. We derive recoverability results that can be used by epidemiologists to obtain or interpret their estimates given the canonical DAG(s) they consider plausible in their study. The article is organized as follows. First, we describe the canonical DAGs. Second, we determine the recoverability of parameters of major interest in each DAG. Third, we investigate the performance of available-case and multiple-imputation procedures. Finally, we use data from the Longitudinal Study of Australian Children (LSAC) to illustrate how our findings can be applied.

## CANONICAL CAUSAL DIAGRAMS

We consider a general point-exposure study with incomplete exposure (X), incomplete outcome (Y), a set of complete confounders $\left(Z_{1}\right)$, and a set of incomplete confounders $\left(Z_{2}\right)$. All of these variables can be of any type (binary, continuous, etc.), and $Z_{1}$ and $Z_{2}$ can be univariate or multivariate.

### Illustrative example

Our example examines the association between maternal mental illness and child behavior based on 4,882 children from the LSAC kindergarten cohort (17): children aged 4–5 years recruited in 2004 (wave 1 of LSAC; approved by the Australian Institute of Family Studies Ethics Committee), with five 2-yearly follow-up waves. The exposure variable (*X*) was a binary indicator of probable serious mental illness at wave 1 (yes/no; 15% missing), designated as affirmative if the mean value across the 6 items of the Kessler Psychological Distress Scale (18) was less than 4. The outcome variable (*Y*) was the child’s score on the Strengths and Difficulties Questionnaire (SDQ) (range, 0–40; 23% missing) at wave 3. A higher score indicates increased behavioral difficulties.

Several potentially confounding covariates relating to the child, mother, and family were measured at wave 1. The completely observed covariates $\left(Z_{1}\right)$ were: sex of child; whether the child had siblings (yes/no); maternal completion of high school (yes/no); maternal age (years); consistent parenting score (range, 1–5); family financial hardship score (range, 0–6); and child’s SDQ score at wave 1. The incomplete covariates $\left(Z_{2}\right)$ were: maternal current smoking status (yes/no; 16% missing); maternal risky alcohol drinking (>2 standard alcoholic drinks per day (yes/no); 18% missing); and child’s physical functioning score (Pediatric Quality of Life Inventory (range, 0–100); 15% missing). Overall, 19% of the children had any covariate in $Z_{2}$ missing, and 34% had any variable in $\left(X,Y,Z_{2}\right)$ missing.

### Canonical complete-data DAG

A causal DAG depicts assumptions about causal relationships between variables using nodes connected by directed arrows (19, 20). The omission of variables or arrows encodes assumptions about the absence of relationships. With missing data, we first consider the DAG that would be assumed if the data were complete: the complete-data DAG (c-DAG).

**Figure 1.**
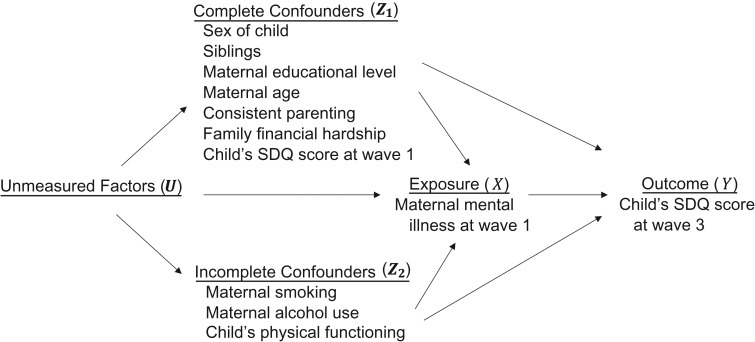
Canonical complete-data directed acyclic graph (c-DAG) for a general point-exposure study. For illustration, we provide under each node heading the variables involved in an example study of maternal mental illness and child behavior that used data from waves 1–3 of the Longitudinal Study of Australian Children (2004–2008). SDQ, Strengths and Difficulties Questionnaire.

Figure 1 shows the “canonical” c-DAG of a point-exposure study like the one in our LSAC example, where certain simplifications are used as a diagrammatic shorthand to encode, in a general way, the “no unmeasured or residual confounding” assumption usually underlying the primary analysis of such studies. To represent that the set of measured covariates is a sufficient set for confounding adjustment, a vector ***U*** representing all completely unmeasured common causes of the exposure and outcome is included. Further, the relationships between the measured covariates themselves are not depicted; rather, all covariates have been collected into 2 potentially vector-valued nodes representing the variables that are completely $\left(Z_{1}\right)$ or incompletely $\left(Z_{2}\right)$ observed.

### Canonical missingness DAGs

Mohan et al. (9, 13–15) defined a “missingness graph,” referred to here as a missingness DAG (m-DAG), as an extension of the c-DAG including the variable-specific missingness indicators to depict assumptions relating to missingness in each variable. This is more detailed than including the “complete case” indicator (21). The missingness indicator for *X* is defined as $M_{X}=1$ if *X* is missing and $M_{X}=0$ otherwise, and $M_{Y}$ is defined similarly. We consider the incomplete confounders all together, with missingness indicator $M_{Z_{2}}=1$ if any of the components of $Z_{2}$ is missing, and $M_{Z_{2}}=0$ otherwise. This simplification limits the number of possible DAGs while still capturing the essential detail for most practical purposes.

When constructing the m-DAG, the missingness indicators should be treated like any other variable, with all causal relationships depicted. To contain the complexity in constructing our canonical m-DAGs, we make 4 assumptions. The first 2 assumptions extend the “no unmeasured or residual confounding” assumption to the missingness setting:

- Assumption 1: There are no unmeasured common causes of a c-DAG variable and a missingness indicator. This precludes direct arrows from ***U*** to the missingness indicators and from ***W***, the vector of unmeasured common causes of the missingness indicators, to c-DAG variables.
- Assumption 2: There are no measured common causes of a c-DAG variable and a missingness indicator that are absent from the c-DAG. This precludes consideration of so-called “auxiliary variables” at the stage of making causal assumptions (see Discussion).

The next 2 assumptions are grounded in a truly causal interpretation of the arrows in a DAG, including the consideration that causality requires a cause to temporally precede an effect:

- Assumption 3: There are no direct arrows from missingness indicators to c-DAG variables. Such causal relationships would be plausible only in exceptional settings (e.g., when the data are used to determine a treatment).
- Assumption 4: There are no direct arrows between the missingness indicators. Such causal relationships would be rare in the point-exposure study, since, aside from the outcome, all variables and their missingness indicators are measured at the same time and so cannot cause one another. Similarly, the missingness of a variable at baseline would not truly cause missingness in the outcome a few years later.

Assumptions 1–3 imply that associations between a missingness indicator and a c-DAG variable can arise only in 2 ways: 1) a direct arrow from the substantive variable to the missingness indicator and 2) common causes of the substantive variable and the missingness indicator among c-DAG variables. By assumptions 1, 2, and 4, associations between missingness indicators can arise from either common causes among c-DAG variables or unmeasured common causes ***W*** distinct from ***U***. In the Discussion, we elaborate on the possibility of relaxing these assumptions.

**Figure 2.**
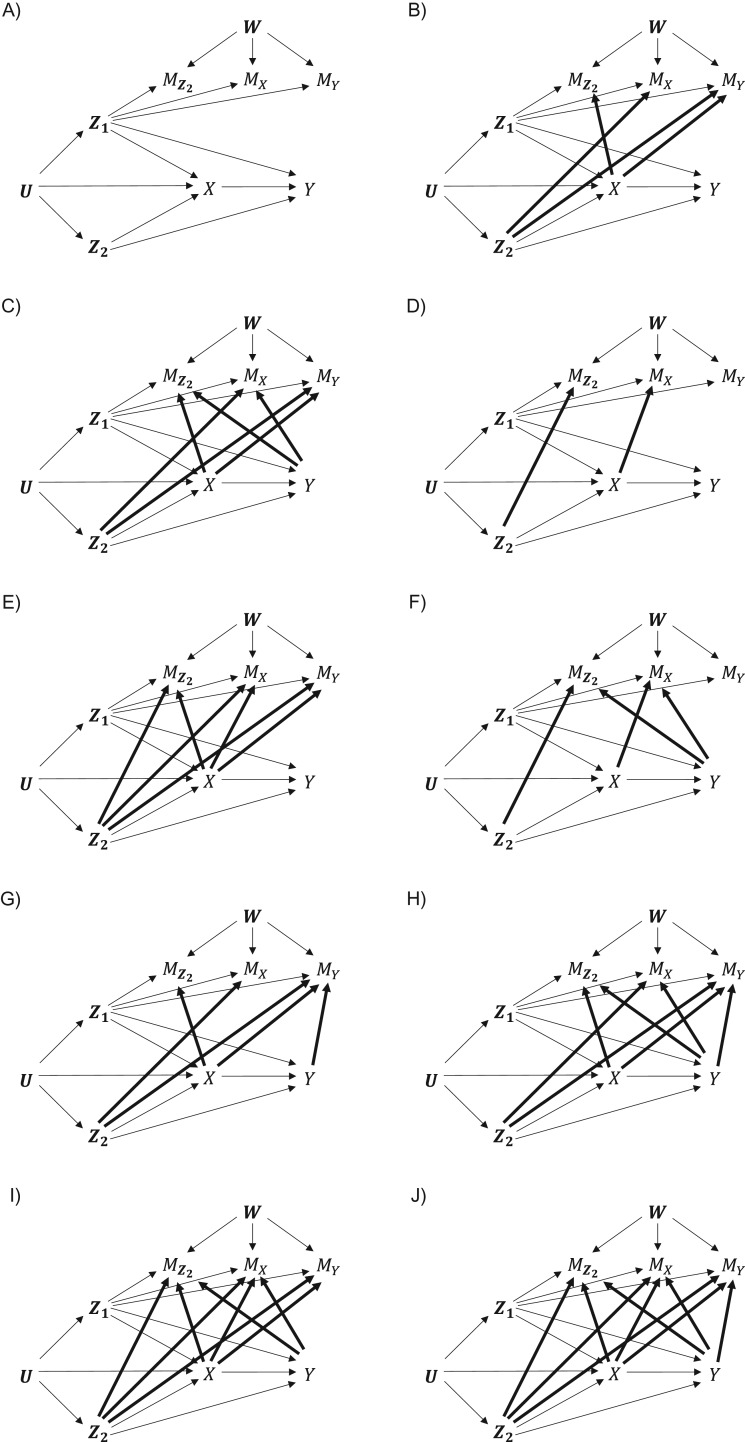
Canonical missingness directed acyclic graphs (m-DAGs) for a general point-exposure study. These 10 m-DAGs were identified as providing the most general forms of all essentially distinct extensions of the m-DAG shown in panel A (referred to as “m-DAG A”) in terms of recoverability. To illustrate how each m-DAG extends m-DAG A, the additional arrows are indicated with a heavier line. In the text and tables, we refer to each m-DAG according to its figure locant (m-DAG A, m-DAG B, etc.).

Assumptions 1–4 limit the number of possible m-DAGs extending the c-DAG. Following a process summarized here and detailed in Web Appendix 1 (available at https://academic.oup.com/aje), we identified 10 m-DAGs that provide the most general forms of all essentially distinct extensions of m-DAG A in Figure 2 in terms of recoverability (see next section). This case forms the starting point, since it assumes the existence of arrows from completely observed confounders $\left(Z_{1}\right)$ to the missingness indicators $\left(M_{Z_{2}},M_{X},M_{Y}\right)$, as is highly plausible in most epidemiologic studies. Briefly, we classified all extensions of m-DAG A into 16 categories according to whether there were arrows from 1) confounders and/or the exposure variable to missingness indicators of other variables, 2) confounders and/or the exposure variable to their own missingness indicators, 3) the outcome variable to missingness indicators of other variables, and 4) the outcome variable to its own missingness indicator. The m-DAG with the most arrows was selected as the “canonical” representative of each class, since it is the most general. Ten of the resulting 16 canonical m-DAGs, shown in Figure 2, were selected because they represent all distinct recoverability scenarios while having the most arrows.

## RECOVERABILITY OF TARGET PARAMETERS

### Definition of recoverability

Regardless of variable type (binary, continuous, etc.), 3 common target parameters in point-exposure studies are: 1) the expected value of the exposure (e.g., mean, proportion); 2) the expected value of the outcome; and 3) the exposure-outcome association adjusted for confounding through regression (e.g., regression-adjusted mean difference or odds ratio). Researchers would like to recover the estimates of these quantities that they would have obtained had there been no missing data. A parameter is recoverable if, based solely on causal assumptions, its value can be expressed as a function of the (large-sample) distribution of the available data (9, 13–15). In more formal statistical terms, recoverability is the same as nonparametric identifiability, meaning that it is possible to consistently estimate the parameter from the available data using an appropriate procedure. In Web Appendix 2, we provide a formal definition of recoverability by defining estimands in terms of potential outcomes.

One can similarly define the recoverability of target distributions. The 3 aforementioned target parameters are characteristics of the marginal exposure and outcome distributions and the conditional outcome distribution given $X,\;Z_{2}$, and $Z_{1}$, respectively. The recoverability of these distributions implies the recoverability of the corresponding parameters. If the joint distribution of $Y,\;X,Z_{2}$, and $Z_{1}$ is recoverable, then all target distributions and parameters are recoverable.

### Conditions for recoverability

In Web Appendix 2, we describe general conditions that are required for recoverability. These broadly mirror those required for causal effect estimation (22), but the multivariable missingness situation is considerably more complex. In particular, while theoretical results establish sufficient or necessary graphical criteria for recoverability in particular cases, especially for the joint distribution (9, 13–15, 23), currently no algorithm can definitively ascertain the recoverability of a specific parameter given an arbitrary m-DAG (16). Thus, recoverability needs to be ascertained mathematically on a case-by-case basis.

### Recoverability in the point-exposure study

Using results from Mohan et al. (13, 15) and new derivations, we established the recoverability of the joint distribution and the 3 aforementioned target distributions and corresponding parameters in the 10 canonical m-DAGs (Tables 1 and 2). In brief, when no c-DAG variable causes its own missingness, the joint distribution and thus all parameters are recoverable. Otherwise, some quantities are recoverable but others are not. The expectation of a variable that causes its own missingness is nonrecoverable, and neither is the regression-adjusted association if the outcome causes its own missingness. Notably, if a parameter is recoverable in a canonical m-DAG, it is also recoverable in all m-DAGs in the class(es) it represents, since these are obtained by removing arrows (lemma 4 in the paper by Mohan et al. (13)). However, the converse does not hold.

**Table 1.** Recoverability Results for the Missingness Directed Acyclic Graphs (m-DAGs) in Figure 2, Stating Whether Each Distribution and Parameter Was Found to Be Recoverable in Each m-DAG^a^

Missingness DAG	Joint Distribution of $Y,X,Z_{1},Z_{2}$	Marginal Distribution of X		Marginal Distribution of Y		Conditional Distribution of Y	
		Entire Distribution	Expectation (e.g., Proportion Exposed)	Entire Distribution	Expectation (e.g., Mean of Y)	Entire Distribution	Expectation (If Yes, Also Holds for the Regression Coefficient)
A	Yes^b^	Yes^b^	Yes	Yes^b^	Yes	Yes^b^	Yes
B	Yes^c^	Yes^d^	Yes	Yes^d^	Yes	Yes^b^	Yes
C	Yes^c^	Yes^d^	Yes	Yes^d^	Yes	Yes^d^	Yes
D	No^e^	No^e^	No	Yes^b^	Yes	Yes^b^	Yes
E	No^e^	No^e^	No	Unable to establish	Conjecture no unless $M_{Y}⊥\;M_{Z_{2}},M_{X}|Z_{1},\;Z_{2},X$^b^	Yes^b^	Yes
F	No^e^	No^e^	No	Yes^b^	Yes	Unable to establish	Conjecture no^b^
G	No^e^	Yes^d^	Yes	No^e^	No	No^f^	No
H	No^e^	Unable to establish	Conjecture no unless $M_{X}⊥\;M_{Z_{2}},M_{Y}|Z_{1},\;Z_{2},Y$^b^	No^e^	No	No^f^	No
I	No^e^	No^e^	No	Unable to establish	Conjecture no unless $M_{Y}⊥\;M_{Z_{2}},M_{X}|Z_{1},\;Z_{2},X$^b^	Unable to establish	Conjecture no^b^
J	No^e^	No^e^	No	No^e^	No	No^f^	No

Abbreviation: DAG, directed acyclic graph.

^a^ Expressions in terms of available data provided in Table 2 in case of recoverability.

^b^ Proof is provided in Web Appendix 2.

^c^ Result obtained by corollary 1 in the paper by Mohan and Pearl (15).

^d^ By recoverability of joint distribution (possibly of a reduced graph).

^e^ By theorem 3 in the paper by Mohan and Pearl (15).

^f^ By corollary 2 in the paper by Mohan and Pearl (15).

**Table 2.** Recoverability Results for the Missingness Directed Acyclic Graphs in Figure 2, Providing for Each Recoverable Distribution Its Mathematical Expression in Terms of Available Data^a,b,c^

Missingness DAG	Joint Distribution	Marginal Distribution of X	Marginal Distribution of Y	Conditional Distribution of Y
A	$P\left(Y,X,Z_{2}|Z_{1},M=0\right)\times P\left(Z_{1}\right)$	$\underset{Z_{1}}{\sum }P\left(X|Z_{1},M_{X}=0\right)\times P\left(Z_{1}\right)$	$\underset{Z_{1}}{\sum }P\left(Y|Z_{1},M_{Y}=0\right)\times P\left(Z_{1}\right)$	$P\left(Y|X,\;Z_{1},Z_{2},M=0\right)$
B	$\frac{P\left(Y,X,\;Z_{2},Z_{1},M=0\right)}{\begin{array}{c} P\left(M_{Y}=0|Z_{1},Z_{2},X,\;M_{Z_{2}}=0,M_{X}=0\right)\\ \times P\left(M_{Z_{2}}=0|Z_{1},X,\;M_{X}=0\right)\\ \times P\left(M_{X}=0|Z_{1},Z_{2},\;M_{Z_{2}}=0\right) \end{array}}$	No simple expression	No simple expression	$P\left(Y|X,\;Z_{1},Z_{2},M=0\right)$
C	$\frac{P\left(Y,X,\;Z_{2},Z_{1},M=0\right)}{\begin{array}{c} P\left(M_{Y}=0|Z_{1},Z_{2},X,\;M_{Z_{2}}=0,M_{X}=0\right)\\ \times P\left(M_{Z_{2}}=0|Z_{1},X,Y,\;M_{X}=0,M_{Y}=0\right)\\ \times P\left(M_{X}=0|Z_{1},Z_{2},Y,\;M_{Z_{2}}=0,M_{Y}=0\right) \end{array}}$	No simple expression	No simple expression	No simple expression
D			$\underset{Z_{1}}{\sum }P\left(Y|Z_{1},M_{Y}=0\right)\times P\left(Z_{1}\right)$	$P\left(Y|X,Z_{1},Z_{2},M=0\right)$
E				$P\left(Y|X,Z_{1},Z_{2},M=0\right)$
F			$\underset{Z_{1}}{\sum }P\left(Y|Z_{1},M_{Y}=0\right)\times P\left(Z_{1}\right)$	
G		No simple expression		
H, I, J				

Abbreviation: DAG, directed acyclic graph.

^a^ Proofs are provided in Web Appendix 2.

^b^ A blank space is left where the distribution is not recoverable or it has not been established as documented in Table 1.

^c^***M*** = (*M_Y_*, *M_X_* , *M**_Z_2__***), **0** = (0, 0, 0).

## ESTIMATION WITH COMMON MISSING-DATA METHODS

### Estimation of recoverable parameters

By definition, recoverable parameters can be consistently estimated on the basis of available data only using an appropriate method. Theoretically, “maximally efficient” estimation requires semiparametric methods that have been investigated in specific settings (24–27), but we do not consider those here, as they require tailoring for each m-DAG and recoverable parameter and have not commonly been used. Instead, we consider the performance, from a theoretical standpoint, of 2 common approaches that are readily implementable for any m-DAG and parameter: “available-case analysis” and multiple imputation.

Available-case analysis consists of estimating the target parameter using only records with complete data on the variables involved (e.g., those with $M_{X}=0$ for estimating the expectation of *X*). For the regression-adjusted exposure-outcome association, this approach coincides with “complete-case analysis” and is unbiased in m-DAGs A, B, D, and E because the conditional distribution is expressible as the conditional distribution among the complete cases (Table 2). For other recoverable parameters, the available-case analysis could be subject to selection bias.

Multivariate normal imputation (2) and multiple imputation by chained equations (MICE) (1) are the most common multiple imputation approaches for handling multivariable missingness. While the former imposes a normal assumption on the full joint distribution, MICE approximates the joint distribution by iteratively imputing all incomplete variables using conditional models that include all other variables as predictors. These approaches can potentially overcome the selection bias that is expected in some situations with available-case analysis. However, they involve parametric assumptions beyond those of the analysis model (e.g., the outcome regression), which may entail a gain in precision relative to available-case analysis but could also induce misspecification bias. The unbiasedness of multiple imputation largely depends on the quality of the parametric assumptions with respect to the target parameter, which is related to the notion of “congeniality” between the imputation and analysis models (28).

The comparative performance of the two approaches is therefore difficult to establish in general because they are subject to different sources of bias, depending on the causal (m-DAG) and parametric assumptions. Instead, simulation studies are required to assess bias in specific contexts.

### Estimation of nonrecoverable parameters

Nonrecoverability arises from inestimable systematic differences between the observed and missing data. Such differences can induce an insuperable selection bias in both the available-case approach and standard implementations of multiple imputation, but bias magnitudes depend on the context (see next section). Consistent estimation of nonrecoverable parameters requires introducing external data, typically in the form of expert-elicited values for the unknown differences. Given the inherent uncertainty in elicitation, estimation can be framed as a sensitivity analysis, producing a range of estimates for the target parameter corresponding to expert-elicited ranges of values for a set of sensitivity parameters. Typically, the analysis includes the setting in which all sensitivity parameters are 0.

An extended discussion on sensitivity analyses is beyond the scope of this paper, but we note that in setting up such analyses, identification of the parameters that require elicitation can be guided by the m-DAG and recoverability results, since these highlight the problematic arrows that lead to nonrecoverability. As a simple example, nonrecoverability of the exposure expectation in m-DAG D arises from the arrow $X\to M_{X}$, which flags exposure distribution differences between persons with observed exposure data and those with missing exposure data. These differences cannot be estimated from available data and thus would require elicitation. Methods for integrating elicited values in the estimation have been proposed, particularly within the multiple-imputation framework (29–34).

### Findings from simulation study

We conducted a simulation study to investigate bias magnitudes in available-case and MICE analyses across the canonical m-DAGs in a setting like LSAC (Web Appendix 3). Our data-generating mechanism assumed main-effects models for the missingness indicators and set values of inestimable parameters, relating to the effect of a variable on its missingness indicator, to be of a similar magnitude to other associations observed in LSAC. We used MICE with an approximately congenial procedure.

Web Figure 1 provides an overview of the results, which we summarize here. For recoverable parameters, the available-case approach exhibited greater bias for mean values than for association parameters and was approximately unbiased for the latter where expected (m-DAGs A, B, D, and E). Multiple imputation yielded approximately unbiased mean and association estimates, with the latter being more precise than those obtained with the available-case approach. These findings are consistent with the common observation in practice that both approaches yield similar association estimates. For nonrecoverable parameters, both approaches generally exhibited nonnegligible bias for the mean value but limited bias for the association parameter, except for m-DAG H when the underlying associations were very strong, agreeing with observations in the literature (35).

## APPLICATION TO LSAC

In applying our findings to guide the treatment of missing data in the LSAC analysis, we first assess which m-DAG(s) are plausible using substantive knowledge (7–9). In LSAC, data on the exposure (*X*) and the incomplete confounders ($Z_{2}$) were collected through questionnaires completed by parents and returned by mail (36). Incompleteness was due to the form not being returned (76% of missing cases for $Z_{2}$ and 96% for *X*) or the question not being answered (remaining cases). Data on the outcome (SDQ score) were collected via a self-completed questionnaire administered during an in-person interview, and parents who failed to complete the questionnaire were asked to return the form by mail (36). Missing SDQ data were due to either attrition in LSAC (54%), failure to return the form (45%), or not answering the specific question (1%). Major reasons for attrition in LSAC are participants opting out or moving away and loss of contact (37).

**Figure 3.**
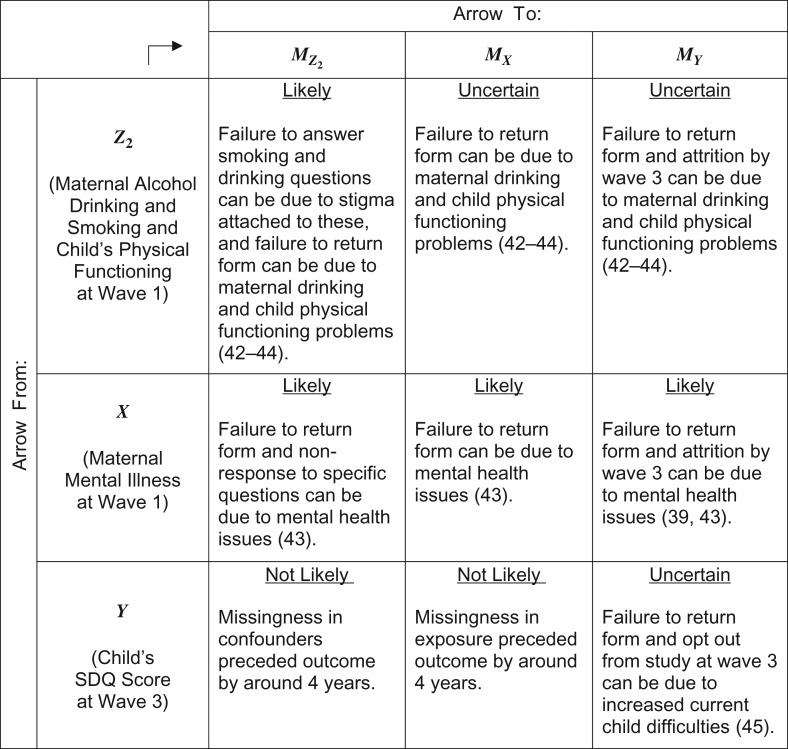
Assessment of the existence of an arrow from each incomplete variable to each missingness indicator in the example from the Longitudinal Study of Australian Children (2004–2008), drawing from evidence in the literature (39, 42–45). SDQ, Strengths and Difficulties Questionnaire.

It is plausible that some complete confounders are causes of missingness in all variables (e.g., low maternal education seems to be a cause of not returning forms and attrition (38, 39)), justifying the choice of m-DAG A as the base scenario. In Figure 3, we document the evidence regarding the possible presence of arrows from each of the incomplete variables to each of the missingness indicators.

Both m-DAG E and m-DAG J appear plausible. In both cases, the proportion exposed is nonrecoverable and sensitivity analyses would be required, and similarly for mean SDQ score, unless we adopt m-DAG E and the additional assumption that missingness in SDQ score is independent of missingness in other variables given the exposure and confounders (Table 1). We do not consider this plausible, since failure to return forms in waves 1 and 3 could have common causes (e.g., behavioral traits) that are not captured by the exposure and confounders. With m-DAG E, the regression-adjusted exposure-outcome association can be unbiasedly estimated using common methods, but sensitivity analyses would be required with m-DAG J.

These remarks shed light on the estimates obtained using available-case analysis and standard MICE with an approximately congenial procedure (Table 3). Both approaches yielded qualitatively similar results for all parameters, and we would expect both methods to be biased for the proportion with maternal mental illness and the mean SDQ score. If m-DAG E is adopted, we would expect both methods to be unbiased for the regression-adjusted association, but with m-DAG J, both methods could be biased.

**Table 3.** Estimates of 3 Target Parameters Using 2 Approaches to Handle Missing Data in the Example Study of Maternal Mental Illness and Child Behavior, Longitudinal Study of Australian Children (Waves 1–3), 2004–2008

Parameter	Estimate (SE)	95% CI	Is Estimate Reliable^a^ if We Adopt:	
			m-DAG E?	m-DAG J?
Proportion of mentally ill mothers at wave 1				
Available-case analysis	0.21 (0.01)	0.20, 0.22	No	No
MICE	0.21 (0.01)	0.20, 0.23	No	No
Mean SDQ score^b^ of children at wave 3				
Available-case analysis	7.48 (0.09)	7.31, 7.65	No	No
MICE	7.74 (0.09)	7.57, 7.90	No	No
Regression-adjusted difference in mean SDQ score^c^				
Available-case analysis	0.59 (0.20)	0.20, 0.98	Yes	No
MICE	0.64 (0.21)	0.23, 1.06	Yes	No

Abbreviations: CI, confidence interval; m-DAG, missingness directed acyclic graph; MICE, multiple imputation by chained equations; SDQ, Strengths and Difficulties Questionnaire; SE, standard error.

^a^ This indicates whether the estimate can be considered reliable according to which m-DAG from Figure 2 is adopted, based on the recoverability of the parameter in that m-DAG.

^b^ Range, 0–40. A higher score indicates increased behavioral difficulties.

^c^ Comparing mentally ill mothers with non–mentally ill mothers.

## DISCUSSION

We investigated the use of m-DAGs in an epidemiologic setting; these were proposed by Mohan et al. (9, 13–15) as a new paradigm with which to frame clearer and finer-grained assumptions about missing data than the classical MAR-MNAR framework. Specifically, we constructed a series of “canonical” m-DAGs, providing results that can guide the analysis of point-exposure studies affected by missing data. The study of canonical structures facilitates the use of m-DAGs in practice, which is currently impeded by the complexity of determining the recoverability of parameters.

In addition to providing intuitive tools to depict detailed assumptions, the m-DAG paradigm reveals that it is crucial to draw a distinction between recoverable and nonrecoverable parameters and between missingness assumptions (m-DAG) and estimation procedures (with their potential parametric assumptions). For recoverable parameters, available-case and multiple-imputation procedures are subject to different sources of bias depending on the assumptions made, but both can be approximately unbiased in certain settings, as seen in our simulation study. Meanwhile, estimation of nonrecoverable parameters warrants sensitivity analysis using externally specified parameters. Uncertainty around the m-DAG, and thus recoverability, can have a bigger impact in terms of bias than the choice between estimation approaches. Thus, in settings with more than one plausible m-DAG, the likely recoverability of the target parameter (e.g., across most/almost none of them) should be considered in judging the reliability of estimates derived from different approaches.

Our recoverability results are useful for determining when sensitivity analyses are needed. This is important since these analyses are far from straightforward, requiring access to cooperative experts and elicitation and consensus methods that need to be tailored to each problem (40). Ultimately, the pertinence of undertaking a sensitivity analysis for a parameter hinges on recognizing it as nonrecoverable and assessing the potential magnitude of selection bias, in addition to its relevance for the study. The canonical m-DAGs can also guide sensitivity analyses when they are deemed necessary, which we plan to investigate further in future work.

Our construction of the canonical m-DAGs relied on the treatment of missingness in the confounders taken together, which led to simplified structures. In future work, more detailed DAGs could be considered. Further, the assumptions we made in constructing the canonical m-DAGs may not be appropriate in all contexts. Assumption 1 could be easily violated, similarly to the common occurrence of unmeasured confounding in observational studies. We expect more parameters to be nonrecoverable when there is an unmeasured common cause of a variable and its missingness indicator, since this is similar, from a recoverability point of view, to the situation where there are direct arrows between these.

It would be possible to relax assumption 2, constructing m-DAGs that include auxiliary variables, which are often available in studies such as LSAC. This is a pragmatically driven limitation of our proposal. Given that auxiliary variables usually have missing data themselves, incorporating them into the m-DAGs not only would imply a considerable increase in the number of scenarios but also would require researchers to consider assumptions about missingness in these variables of secondary importance. We are investigating feasible avenues for incorporating auxiliary variables in the “assumptions” step, but in the meantime we suggest that these continue to be used in the “estimation” step in multiple-imputation procedures, as they are usually beneficial for precision (41).

Relaxing assumptions 3 and 4 would not only lead to an increase in the number of scenarios but also require further theoretical work. Assumption 3 underlies all the results of Mohan et al., and the setting in which assumption 4 is relaxed is treated separately by these authors and is substantially more complex mathematically (9, 13–15). Fortunately, these assumptions appear reasonable in the point-exposure design.

Mohan et al. (9, 13–15) proposed DAG-based definitions of “missing completely at random” and “MAR,” which in our study correspond to the “trivial” m-DAG with no arrows from c-DAG variables to missingness indicators (see Web Appendix 1) and m-DAG A, respectively. The connections between the graph-based and classical (4, 5, 11) definitions have been explored (6, 9, 13). The graph-based “MAR” is stronger than (everywhere) MAR, although the two are equivalent under additional conditions: 1) independent records and 2) independence of missingness indicators given substantive variables (i.e., absence of ***W*** in our m-DAGs) (5, 10, 11). Then, MAR implies that missingness can depend only on fully observed variables, becoming more stringent as the number of incomplete variables grows. Understanding these connections is of interest, since the classical definitions underlie the theoretical results that underpin common missing-data methods. However, the full potential of the m-DAG approach is realized when focus is shifted from the MAR-MNAR classification and directed towards the specification of detailed mechanisms, a substantial number of which allow the possibility of unbiased estimation without needing to specify unidentifiable sensitivity parameters.

A limitation of DAGs is the impossibility of portraying interactions. Our m-DAGs thus encode structural assumptions about the missing-data mechanism, that is, main-effects models. These are reflected in our simulation study, which constitutes an initial investigation, under particular conditions, of the performance of common estimation approaches in conjunction with the proposed causal modeling framework. Further simulations are needed to assess biases in more general settings, considering missingness models with interactions, and also comparing performance with semiparametric estimators built, for example, using the recoverability formulae provided in Table 2.

In conclusion, our findings can be used to guide the treatment of missing data in point-exposure studies, and they provide avenues for future work on refining DAGs, estimation, and sensitivity analysis procedures.

## Supplementary Material

Web Material

## Abbreviations

c-DAG: complete-data directed acyclic graph
DAG: directed acyclic graph
LSAC: Longitudinal Study of Australian Children
MAR: missing at random
m-DAG: missingness directed acyclic graph
MICE: multiple imputation by chained equations
MNAR: missing not at random
SDQ: Strengths and Difficulties Questionnaire
