# Correction to: “Canonical causal diagrams to guide the treatment of missing data in epidemiologic studies”

**DOI:** 10.1093/aje/kwae406

**Published:** 2025-01-17

**Authors:** Margarita Moreno-Betancur, Katherine J Lee, Finbarr P Leacy, Julie A Simpson, John B Carlin

**Affiliations:** Clinical Epidemiology and Biostatistics Unit, Murdoch Children’s Research Institute, Melbourne, Australia; Clinical Epidemiology and Biostatistics Unit, Department of Paediatrics, University of Melbourne, Melbourne, Australia; Clinical Epidemiology and Biostatistics Unit, Murdoch Children’s Research Institute, Melbourne, Australia; Clinical Epidemiology and Biostatistics Unit, Department of Paediatrics, University of Melbourne, Melbourne, Australia; Health Products Regulatory Authority, Dublin, Ireland; Centre for Epidemiology and Biostatistics, Melbourne School of Population and Global Health, University of Melbourne, Melbourne, Australia; Clinical Epidemiology and Biostatistics Unit, Murdoch Children’s Research Institute, Melbourne, Australia; Clinical Epidemiology and Biostatistics Unit, Department of Paediatrics, University of Melbourne, Melbourne, Australia

**Keywords:** causal inference, directed acyclic graphs, missing data, missing at random, missing not at random, multiple imputation, recoverability, sensitivity analysis

##  

Moreno-Betancur et al.^1^ report and correct here errors in their article “Canonical causal diagrams to guide the treatment of missing data in epidemiologic studies” published in the *Journal* in 2018.

A subset of the theoretical results provided in Tables 1 and 2 of the original article was incorrect, specifically for the following cases: joint and marginal distributions for missingness directed acyclic graph (m-DAG) B, all distributions for m-DAG C, and marginal exposure distribution for m-DAG G. Each of these distributions (and their expectations) are, in fact, nonrecoverable. This is shown in new proofs provided in Appendix S1 of the Supplementary Material accompanying this correction, which draw on both prior and more recent work in the field.^2^^-^^6^ These errors arose from the incorrect application of Corollary 1 of Mohan et al.^6^ in the original mathematical proofs. This corollary does not actually apply to m-DAGs B, C, and G, because the presence of unobserved common causes $\left(\boldsymbol{W}\right)$ of the missingness indicators induces what Mohan et al.^6^ call “collider paths,” a terminology that Moreno-Betancur et al. had, unfortunately, misinterpreted. Importantly, the corrected results for m-DAGs B and C imply that, contrary to what was stated when describing the theoretical results in the original article,^1^ some distributions may not be recoverable even when no substantive variable causes its own missingness.

Further implications of these corrected recoverability results are as follows. First, the corrected results imply the nonrecoverability of the marginal outcome distribution in m-DAGs E and I, the marginal exposure distribution in m-DAG H, and the conditional outcome distribution in m-DAG I (proofs in Appendix S2), which had been left as open questions in the original article. Additionally, the techniques used to prove the corrected results provided a way to prove the nonrecoverability of the marginal exposure distribution in m-DAG H2 and the conditional outcome distribution in m-DAG F (proofs in Appendix S2), which had also been left as open questions in the original article. Notably, the interpretations of the simulation study and illustrative example had been based on the conjecture that, unless perhaps further implausible assumptions were made, the expectations of the distributions in question were nonrecoverable, which is now confirmed.

Second, contrary to what had been stated in Web Appendix 1 of the original article, recoverability results differ between m-DAGs C and C2, and between m-DAGs G and G2. Results for m-DAGs C2 and G2 remain correct, but they no longer follow from results for m-DAGs C and G. New proofs of the results for m-DAGs C2 and G2 are provided in this correction (Appendix S3). This also means that the 10 m-DAGs presented in Figure 2 of the original article no longer represent all distinct recoverability scenarios among all 16 canonical m-DAGs, as had been stated in the original article.^1^ For clarity, in Figures 1 and 2 of this correction, Moreno-Betancur et al. present complete and correct recoverability results for all 16 canonical m-DAGs, highlighting those results that have been corrected or newly established because of the correction.

**Figure 1:**
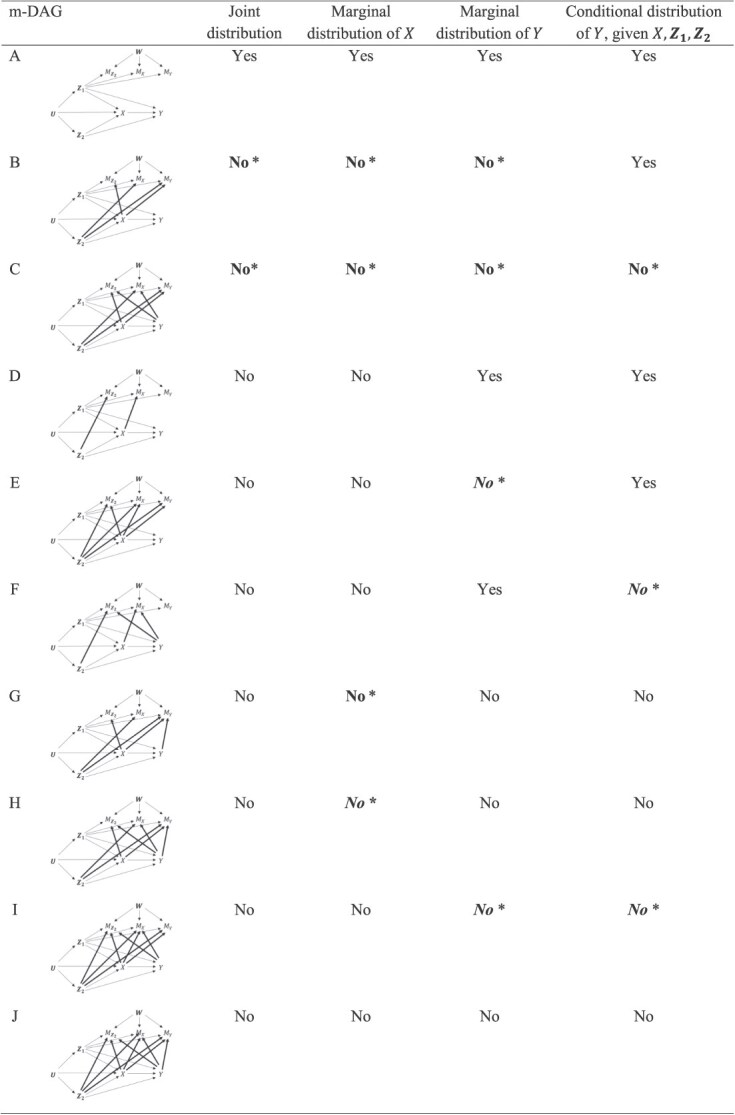
Recoverability (yes/no) of distributions for the 10 canonical missingness directed acyclic graphs (m-DAGs) that were the focus in the main text of the original manuscript. Regular font indicates results that have not changed; **bold regular** font indicates results that have changed because of this correction; and ***bold italic*** font indicates results that have been newly established because of this correction. An asterisk (*) indicates that the proof of this result is provided in the Supplementary Material accompanying this correction. Correct and detailed proofs for all other results (ie, those without an asterisk) were provided in the original article.

**Figure 2:**
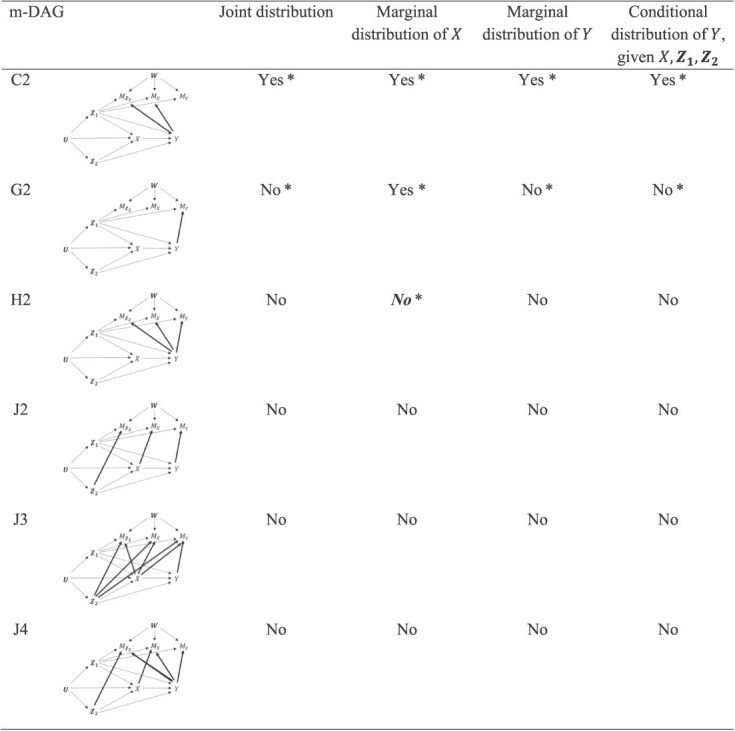
Recoverability (yes/no) of distributions for the 6 canonical missingness directed acyclic graphs (m-DAGs) that appeared in Web Appendix 2 of the original article. Regular font indicates results that have not changed and ***bold italic*** font indicates results that have been newly established because of this correction. An asterisk (*) indicates that the proof of this result is provided in the Supplementary Material accompanying this correction (in some cases, for a result that is unchanged but no longer follows from proofs provided in the original article). Correct and detailed proofs for all other results (ie, those without an asterisk) were provided in the original article.

Finally, revisiting the interpretation of the simulation findings in the original article and its accompanying Web Appendix 3 in light of the corrected and newly established results, it can be observed that multiple imputation provided approximately unbiased estimates of nonrecoverable parameters in a range of settings in which available case analysis estimates had non-negligible bias, most obviously when the underlying associations were strong (exposure mean: m-DAGs B, C, G, H; outcome mean: m-DAGs B, C, E; association parameter: m-DAGs C, I). Whether this observation generalizes to settings beyond these simulation scenarios is a matter for future research.

Further minor corrections in the Web Material of the original paper are as follows. On page 4, under “Target Parameters and Target Distributions,” there was a typographical error: the second term of the displayed expression at the bottom should read ${X}^{\boldsymbol{M}=\mathbf{0}}=0$ instead of ${X}^{\boldsymbol{M}=\mathbf{0}}=1$. On page 8, the last line of the paragraph on “Failure of IPW-Type Identification” is incorrect because $Y$ and ${M}_X$ and ${M}_{\boldsymbol{Z}\mathbf{2}}$ are not conditionally independent, given the other variables. This derivation is now superfluous, given the proofs provided in this correction of the nonrecoverability of the conditional outcome distribution in m-DAGs F and I. On page 12, line 3, there was a typographical error: “The bias was non-negligible in Scenario 3 for the proportion exposed and the regression coefficient in m-DAG D” should have read “in m-DAG C.”

The authors regret these errors.

## Supplementary Material

Web_Material_kwae406
