# Supplementary material for: Canonical Causal Diagrams to Guide the Treatment of Missing Data in Epidemiologic Studies
Source: Am J Epidemiol. 2018 Aug 14;187(12):2705–15. doi: 10.1093/aje/kwy173 (PMC6269242; doi:10.1093/aje/kwy173)
Supplement: Web Material [file kwy173_moreno-betancur_webmaterial_final.pdf]

Web Material for

"Canonical Causal Diagrams to Guide the Treatment of Missing Data in Epidemiologic Studies"

Margarita Moreno-Betancur, Katherine J. Lee, Finbarr P. Leacy, Ian R. White, Julie A. Simpson, and John B. Carlin

The contents of this document are as follows:

- **Web Appendix 1** details the construction of the canonical m-DAGs, provides further recoverability results and presents a study of the "trivial" m-DAG.
- **Web Appendix 2** provides technical material on recoverability, including its definition and conditions for recoverability based on a potential outcomes approach, and recoverability proofs for results in Tables 1 and 2 of the main text.
- **Web Appendix 3** presents a simulation study, based on LSAC, investigating the performance of available case and multiple imputation approaches for estimation of the three target parameters across all m-DAGs under certain conditions.
- **Web Tables 1–5**
- **Web Figures 1–3**
- **References**

Notation and abbreviations are as in the main text.

WEB APPENDIX 1: Construction of the canonical m-DAGs, recoverability results and the "trivial" m-DAG

Classes of m-DAGs and canonical representatives

The canonical m-DAGs studied throughout the paper were constructed by considering all the possible extensions of m-DAG A in Figure 2 of the main text. Under Assumptions 1-4 in the main text, the number of extensions of m-DAG A is equal to $2^9 = 512$, which is calculated by considering all the possible combinations of presence or absence of 9 arrows, from each of the variables $\boldsymbol{Z_2}, X$ and Y to each of the missingness indicators $M_{\boldsymbol{Z_2}}, M_X$ and M_Y. We classified these m-DAGs according to four characteristics that are broadly important for the recoverability of the joint and outcome distributions, the latter being the main focus in point-exposure studies. Specifically, we classified them according to the presence of arrows

- from $\boldsymbol{Z_2}$ and/or X to the missingness indicators of other variables, i.e. from $\boldsymbol{Z_2}$ to M_X and/or M_Y and/or from X to $M_{\boldsymbol{Z_2}}$ and/or M_Y (Yes/No),
- from $\boldsymbol{Z_2}$ and/or X to their own missingness indicators, i.e. from $\boldsymbol{Z_2}$ to $M_{\boldsymbol{Z_2}}$ and/or from X to M_X (Yes/No),
- from Y to the missingness indicators of other variables, $M_{\boldsymbol{Z_2}}$ and/or M_X, (Yes/No), and
- from Y to its own missingness indicator M_Y (Yes/No).

Considering possible combinations of answers to these four questions leads to $2^4 = 16$ broad classes of m-DAGs. We selected as "canonical" representative of each class the m-DAG corresponding to the case where all the "and/or" above are replaced by "and" whenever the answer was "Yes". Hence the canonical m-DAG is the most general m-DAG in that class in terms of having the most arrows. Ten out of the sixteen canonical m-DAGs are presented in Figure 2 in the main text, and the remaining 6 are presented in Web Figure 2 in this document (produced using the R package "dagitty" [1]). Below we describe how the ten m-DAGs in Figure 2 were selected, but first we discuss recoverability in a class of m-DAGs.

Recoverability in canonical m-DAG versus other m-DAGs in a class

The canonical m-DAG of a class, being the most general, is the one where recoverability of parameters is less likely. Indeed, if a parameter is recoverable in the class's canonical m-DAG, then it is recoverable in all the m-DAGs in the class – this is proved formally in Lemma 4 of [2]. Note however that the converse does not hold, meaning that the results for the canonical m-DAG are the most pessimistic for the class.

Recoverability results for canonical m-DAGs

Recoverability results for m-DAGs A-J in the main text are presented in Table 1 of the main text. These ten m-DAGs were selected as they represent all key recoverability scenarios, while being the most general (with the most arrows). Indeed, as suggested by the letter designations of the six remaining m-DAGs in Web Figure 2, recoverability in each of those is the same as in one of the ten m-DAGs in the main text. Specifically:

- For m-DAG C2, recoverability results are the same as for m-DAG C, except that the recoverability expression for the marginal distribution of Y is simpler (as for m-DAG A). We selected m-DAG C as it is the most general.
- For m-DAG G2, recoverability results are the same as for m-DAG G, except that the recoverability expression the marginal distribution of X is simpler (as for m-DAG A). We selected m-DAG G as it is the most general.
- For m-DAG H2, recoverability results are the same as for m-DAG H.
  We selected m-DAG H as it is the most general.
- For m-DAGs J2-J4, recoverability results are the same as for m-DAG J.
  We selected m-DAG J as it is the most general.

The “trivial” m-DAG

Due to its correspondence with the “missing completely at random” assumption, we present recoverability results for the “trivial” m-DAG with no arrows from c-DAG variables to the missingness indicators, not even from the completely observed confounders (Web Figure 3).

In the “trivial” m-DAG all the distributions are recoverable from the available cases, as follows:

- Joint distribution: $P(Y, X, \boldsymbol{Z_1}, \boldsymbol{Z_2} | \boldsymbol{M} = \boldsymbol{0})$
- Marginal distribution of X: $P(X | M_X = 0)$
- Marginal distribution of Y: $P(Y | M_Y = 0)$
- Conditional distribution of Y: $P(Y | X, \boldsymbol{Z_1}, \boldsymbol{Z_2}, \boldsymbol{M} = \boldsymbol{0})$

Thus, the available case approach is unbiased for all parameters in this m-DAG.

WEB APPENDIX 2: Technical material on recoverability

Potential outcomes to express targets

A *target parameter* is some aspect of the joint distribution of c-DAG variables in a counterfactual world without missing data. This is expressible in terms of "potential outcomes":

$$X^{M_Y=0,M_X=0,M_{Z_2}=0} = \text{Value of } X \text{ in world without missing } Y, X, \boldsymbol{Z_2},$$

$$Y^{M_Y=0,M_X=0,M_{Z_2}=0} = \text{Value of } Y \text{ in world without missing } Y, X, \boldsymbol{Z_2}, \text{ and}$$

$$\boldsymbol{Z}_{\boldsymbol{2}}^{M_Y=0,M_X=0,M_{Z_2}=0} = \text{Value of } \boldsymbol{Z_2} \text{ in world without missing } Y, X, \boldsymbol{Z_2}.$$

We will also use the notation $Y^{\boldsymbol{M=0}}, X^{\boldsymbol{M=0}}, \boldsymbol{Z}_{\boldsymbol{2}}^{\boldsymbol{M=0}}$ where $\boldsymbol{M} = (M_Y, M_X, M_{\boldsymbol{Z_2}})$ and $\boldsymbol{0} = (0,0,0)$. Thus, the superscript $\boldsymbol{M} = \boldsymbol{0}$ is used to indicate the value of the superscripted variable if, possibly contrary to the fact, none of the variables were missing. This conceptualization of the problem means that the missingness indicators are viewed as "treatments" on which we can potentially intervene to switch them on and off. This is similar to the treatment of censoring by Hernan and Robins [3].

Of note, one could also express some parameters in terms of potential outcomes in intermediate worlds where the missingness in only one or two variables is precluded. For example $Y^{M_Y=0}$, which is the value of Y in a world without missing, could be used to express the target marginal distribution of the outcome. This choice would require different consistency assumptions for different parameters, rather than the global set that we provide further below. We feel that this complicates matters, perhaps unnecessarily, and thus chose to express all target parameters and distributions in relation to a world in which none of the variables are missing, as follows.

Target parameters and target distributions

The target parameters considered in the manuscript are expressible using the potential outcomes as follows. The expectation of the exposure (e.g. means, proportions) is $E(X^{\boldsymbol{M=0}})$**,** the expectation of the outcome is $E(Y^{\boldsymbol{M=0}})$, and, for a binary 1/0 exposure, the coefficient of the exposure in generalized linear regression model of Y on X, $\boldsymbol{Z_2}, \boldsymbol{Z_1}$ (e.g. regression-adjusted mean difference, log-odds ratio, log-risk ratio) is given by:

$$g\{E(Y^{\boldsymbol{M=0}}|X^{\boldsymbol{M=0}} = 1, \boldsymbol{Z}_{\boldsymbol{2}}^{\boldsymbol{M=0}}, \boldsymbol{Z_1})\} - g\{E(Y^{\boldsymbol{M=0}}|X^{\boldsymbol{M=0}} = 1, \boldsymbol{Z}_{\boldsymbol{2}}^{\boldsymbol{M=0}}, \boldsymbol{Z_1})\}$$

where g is the link function in the generalized linear model (e.g. identity for linear regression, logit for logistic regression).

These three parameters are specific characteristics of the following target probability density distributions: $P(X^{\boldsymbol{M=0}})$, $P(Y^{\boldsymbol{M=0}})$ and $P(Y^{\boldsymbol{M=0}}|X, \boldsymbol{Z_2}, \boldsymbol{Z_1})$. These are in turn aspects of the target joint distribution of all variables, denoted by $P(Y^{\boldsymbol{M=0}}, X^{\boldsymbol{M=0}}, \boldsymbol{Z}_{\boldsymbol{2}}^{\boldsymbol{M=0}}, \boldsymbol{Z_1})$.

Definition of recoverability

A target parameter $\theta = \psi\{P(Y^{\boldsymbol{M=0}}, X^{\boldsymbol{M=0}},\ \boldsymbol{Z}_{\boldsymbol{2}}^{\boldsymbol{M=0}}, \boldsymbol{Z}_{\boldsymbol{1}})\}$ is *recoverable* if it can be expressed as a function of the distribution of the observed, incomplete data O. i.e.

$$\theta \text{ is recoverable if } \exists \psi^* \text{ function} : \theta = \psi^*\{P(O)\}.$$

This means that the parameter can be re-expressed as a characteristic of the distribution of the data observed in this world. In other words, a parameter is recoverable if the distribution of the incomplete data observed in this world is consistent with *a single value* of the target parameter in the counterfactual world without missing data. Recoverability is thus simply non-parametric identifiability, i.e. identifiability that relies solely on causal or structural assumptions and not on parametric assumptions.

Of note, the three target parameters defined in the previous section are recoverable if the corresponding target probability distributions are recoverable, and any parameter is recoverable if the target joint distribution is recoverable.

Conditions for recoverability of a target parameter

The conditions required for the recoverability of a target parameter are similar to those required for identification of causal effects [3], except more complicated for several reasons. First, the potential outcomes defined above involve not one but three variables on which we could intervene ($M_Y, M_X, M_{\boldsymbol{Z}_2}$). Second, there are many potential target parameters θ that could be of interest, rather than just a simple treatment group contrast. Third, although we only care about the switch of each indicator from missing (1) to non-missing (0), there are 7 non-trivial missingness patterns. The required conditions are:

1. ***Consistency and well-defined interventions***

- Observed values coincide with those in the counterfactual worlds:
  - $Y^{\boldsymbol{M=0}} = Y$ for those with $M_Y = 0$
  - $X^{\boldsymbol{M=0}} = X$ for those with $M_X = 0$
  - $\boldsymbol{Z}_{\boldsymbol{2}}^{\boldsymbol{M=0}} = \boldsymbol{Z}_{\boldsymbol{2}}$ for those with $M_{\boldsymbol{Z}_2} = 0$
- The intervention that would force everyone to have complete data is well-defined (e.g. missing data is not due to death).

2. ***Positivity***

Given any possible variable combination, the probability of observing a complete case with that combination is positive. That is, for all $(x, y, \boldsymbol{z}_{\boldsymbol{1}}, \boldsymbol{z}_{\boldsymbol{2}})$ for which $P(X^{\boldsymbol{M=0}} = x, Y^{\boldsymbol{M=0}} = y, \boldsymbol{Z}_{\boldsymbol{1}} = \boldsymbol{z}_{\boldsymbol{1}}, \boldsymbol{Z}_{\boldsymbol{2}}^{\boldsymbol{M=0}} = \boldsymbol{z}_{\boldsymbol{2}}) > 0$, we have:

$$P(M_X = 0,\ M_Y = 0, M_{\boldsymbol{Z}_2} = 0 \mid X^{\boldsymbol{M=0}} = x, Y^{\boldsymbol{M=0}} = y, \boldsymbol{Z}_{\boldsymbol{1}} = \boldsymbol{z}_{\boldsymbol{1}}, \boldsymbol{Z}_{\boldsymbol{2}}^{\boldsymbol{M=0}} = \boldsymbol{z}_{\boldsymbol{2}}) > 0$$

3. ***Conditional independence conditions***

The causal assumptions depicted by an m-DAG translate into a specific set of conditional independence properties between the variables that are instrumental in determining recoverability. This translation is achieved through a graphical criterion called d-separation

[4]. In practice, ascertaining d-separation is tedious and software such as the R package “dagitty” [1] can be help in this task. Web Table 1 shows the conditional independence properties implied by the m-DAGs of Figure 2 in the main text, which also hold for the potential outcome variables.

For recoverability, we require that the m-DAG implies conditional independence properties that, along with consistency and positivity, enable expression of θ in terms of $P(O)$. Unfortunately, we cannot be more precise than this in a general sense because the specific conditions required for this to be possible vary both across m-DAGs and parameters. This becomes apparent when observing the varying conditional independence properties implied by each of the m-DAGs in Figure 2 of the main text, and their varying ability to yield recoverability of different parameters and distributions (Table 1 of main text). Specific examples of how recoverability was derived using the conditional independence properties are provided in the final section of this appendix.

Therefore, recoverability generally needs to be ascertained mathematically on a case-by-case basis. Some theoretical results from Mohan et al. [2,5–7] are available, but these mostly focus on recoverability of the joint distribution and there is no available algorithm that is both sound and complete (i.e. an algorithm that can ascertain recoverability correctly in all possible settings) [8]. This means that we cannot easily determine recoverability. Therein lies the pragmatic importance of devising “canonical” m-DAGs for specific study designs that allow us to ascertain the recoverability of typical parameters with sufficient generality by making some simplifications.

Proofs for recoverability results in Tables 1 and 2 of the main text

Proving recoverability amounts to deriving an expression for the target parameter or distribution in terms of the distribution of the observed data.

Derivation of expressions provided in Table 2

m-DAG A

- Joint distribution

$$P\left(Y^{\boldsymbol{M}=\boldsymbol{0}}, X^{\boldsymbol{M}=\boldsymbol{0}}, \boldsymbol{Z}_2^{\boldsymbol{M}=\boldsymbol{0}}, \boldsymbol{Z}_1\right) = P\left(Y^{\boldsymbol{M}=\boldsymbol{0}}, X^{\boldsymbol{M}=\boldsymbol{0}}, \boldsymbol{Z}_2^{\boldsymbol{M}=\boldsymbol{0}} | \boldsymbol{Z}_1\right) \times P(\boldsymbol{Z}_1)$$

$$= P\left(Y^{\boldsymbol{M}=\boldsymbol{0}}, X^{\boldsymbol{M}=\boldsymbol{0}}, \boldsymbol{Z}_2^{\boldsymbol{M}=\boldsymbol{0}} | \boldsymbol{Z}_1, \boldsymbol{M}=\boldsymbol{0}\right) \times P(\boldsymbol{Z}_1)$$

(by conditional independence properties)

$$= P(Y, X, \boldsymbol{Z}_2 | \boldsymbol{Z}_1, \boldsymbol{M}=\boldsymbol{0}) \times P(\boldsymbol{Z}_1) \quad \text{(by consistency)}$$

- Marginal distribution of X

$$P(X^{\boldsymbol{M}=\boldsymbol{0}}) = \sum P(X^{\boldsymbol{M}=\boldsymbol{0}} | \boldsymbol{Z}_1) \times P(\boldsymbol{Z}_1)$$

$$= \sum P(X^{\boldsymbol{M}=\boldsymbol{0}} | \boldsymbol{Z}_1, M_X) \times P(\boldsymbol{Z}_1) \quad \text{(by conditional independence properties)}$$

$$= \sum P(X | \boldsymbol{Z}_1, M_X) \times P(\boldsymbol{Z}_1) \quad \text{(by consistency)}$$

- Marginal distribution of Y: proof similar to that of marginal distribution of X

- Conditional distribution of Y:

$P(Y^{\boldsymbol{M}=\boldsymbol{0}}|X^{\boldsymbol{M}=\boldsymbol{0}}, \boldsymbol{Z}_2^{\boldsymbol{M}=\boldsymbol{0}}, \boldsymbol{Z}_1) = P(Y^{\boldsymbol{M}=\boldsymbol{0}}|X^{\boldsymbol{M}=\boldsymbol{0}}, \boldsymbol{Z}_2^{\boldsymbol{M}=\boldsymbol{0}}, \boldsymbol{Z}_1, \boldsymbol{M} = \boldsymbol{0})$ (by conditional independence properties)

$= P(Y|X, \boldsymbol{Z}_2, \boldsymbol{Z}_1, \boldsymbol{M} = \boldsymbol{0})$ (by consistency)

<u>m-DAG B</u>

- Joint distribution: see below
- Conditional distribution of Y: same proof as for this distribution in m-DAG A

<u>m-DAG C</u>

- Joint distribution: see below

<u>m-DAG D</u>

- Marginal distribution of Y: same proof as for this distribution in m-DAG A
- Conditional distribution of Y: same proof as for this distribution in m-DAG A

<u>m-DAG E</u>

- Conditional distribution of Y: same proof as for this distribution in m-DAG A

<u>m-DAG F</u>

- Marginal distribution of Y: same proof as for this distribution in m-DAG A

Expressions for joint distribution in m-DAGs B and C

Applying Corollary 1 of [9], we can express the joint distribution in m-DAG B in terms of the observed data as follows:

$$P(Y^{\boldsymbol{M}=\boldsymbol{0}}, X^{\boldsymbol{M}=\boldsymbol{0}}, \boldsymbol{Z}_2^{\boldsymbol{M}=\boldsymbol{0}}, \boldsymbol{Z}_1) = \frac{P(Y, X, \boldsymbol{Z}_2, \boldsymbol{Z}_1, \boldsymbol{M} = \boldsymbol{0})}{\begin{array}{c} P(M_Y = 0|\boldsymbol{Z}_1, \boldsymbol{Z}_2, X, M_{\boldsymbol{Z}_2} = 0, M_X = 0) \times \\ P(M_{\boldsymbol{Z}_2} = 0|\boldsymbol{Z}_1, X, M_X = 0) \times \\ P(M_X = 0|\boldsymbol{Z}_1, \boldsymbol{Z}_2, M_{\boldsymbol{Z}_2} = 0) \end{array}}$$

By the same result, the joint distribution in m-DAG C is given by:

$$P(Y^{\boldsymbol{M}=\boldsymbol{0}}, X^{\boldsymbol{M}=\boldsymbol{0}}, \boldsymbol{Z}_2^{\boldsymbol{M}=\boldsymbol{0}}, \boldsymbol{Z}_1) = \frac{P(Y, X, \boldsymbol{Z}_2, \boldsymbol{Z}_1, \boldsymbol{M} = \boldsymbol{0})}{\begin{array}{c} P(M_Y = 0|\boldsymbol{Z}_1, \boldsymbol{Z}_2, X, M_{\boldsymbol{Z}_2} = 0, M_X = 0) \times \\ P(M_{\boldsymbol{Z}_2} = 0|\boldsymbol{Z}_1, X, Y, M_X = 0, M_Y = 0) \times \\ P(M_X = 0|\boldsymbol{Z}_1, \boldsymbol{Z}_2, Y, M_{\boldsymbol{Z}_2} = 0, M_Y = 0) \end{array}}$$

Recoverability of conditional expectation of Y in m-DAGs F and I

We conjecture that this parameter is not recoverable because of the collider structures $X \rightarrow M_X \leftarrow Y$ and $\boldsymbol{Z_2} \rightarrow M_{\boldsymbol{Z_2}} \leftarrow Y$. These structures preclude identification of this parameter among the complete cases due to the collider-stratification bias that arises from conditioning on either M_X or $M_{\boldsymbol{Z_2}}$. Further, the collider structure involves both the corresponding missing variable and the outcome, and as we show next, this makes identification impossible through marginal methods of either the g-formula type (as used above, for example, for the marginal distribution of X in m-DAG A) or the inverse probability weighting (IPW) type (as used, for example, for the joint distribution in m-DAG B).

- *Failure of g-formula-type identification*

The conditional expectation is given by:

$$E\left(Y^{\boldsymbol{M=0}} | X^{\boldsymbol{M=0}}, \boldsymbol{Z_2^{M=0}}, \boldsymbol{Z_1}\right) = E\left(Y | X^{\boldsymbol{M=0}}, \boldsymbol{Z_2^{M=0}}, \boldsymbol{Z_1}, M_Y = 0\right)$$

To further decompose this expression following a g-formula-type rationale, we would need Y to be conditionally independent of M_X and/or $M_{\boldsymbol{Z_2}}$ given a subset of variables, but there is no such subset because Y is a parent of both these indicators.

- *Failure of IPW-type identification*

$$\begin{aligned}
&E\left(Y^{\boldsymbol{M=0}} \middle| X^{\boldsymbol{M=0}}, \boldsymbol{Z_2^{M=0}}, \boldsymbol{Z_1}\right) \\
&= E\left(Y^{\boldsymbol{M=0}} \middle| X^{\boldsymbol{M=0}}, \boldsymbol{Z_2^{M=0}}, \boldsymbol{Z_1}, M_Y = 0\right) \\
&= \frac{P\left(M_X = 0, M_{Z_2} = 0 \middle| X^{\boldsymbol{M=0}}, \boldsymbol{Z_2^{M=0}}, \boldsymbol{Z_1}, M_Y = 0\right)}{P\left(M_X = 0, M_{Z_2} = 0 \middle| X^{\boldsymbol{M=0}}, \boldsymbol{Z_2^{M=0}}, \boldsymbol{Z_1}, M_Y = 0\right)} E\left(Y \middle| X^{\boldsymbol{M=0}}, \boldsymbol{Z_2^{M=0}}, \boldsymbol{Z_1}, M_Y = 0\right) \\
&= E\left(\frac{I\left(M_X = 0, M_{Z_2} = 0\right)}{P\left(M_X = 0, M_{Z_2} = 0 \middle| X^{\boldsymbol{M=0}}, \boldsymbol{Z_2^{M=0}}, \boldsymbol{Z_1}, M_Y = 0\right)} \middle| X^{\boldsymbol{M=0}}, \boldsymbol{Z_2^{M=0}}, Z_1, M_Y = 0\right) \\
&\times E\left(Y \middle| X^{\boldsymbol{M=0}}, \boldsymbol{Z_2^{M=0}}, \boldsymbol{Z_1}, M_Y = 0\right) \\
&= E\left(\frac{I\left(M_X = 0, M_{Z_2} = 0\right) \times Y}{P\left(M_X = 0, M_{Z_2} = 0 \middle| X^{\boldsymbol{M=0}}, \boldsymbol{Z_2^{M=0}}, \boldsymbol{Z_1}, M_Y = 0\right)} \middle| X, \boldsymbol{Z_2}, \boldsymbol{Z_1}, M_Y = 0\right)
\end{aligned}$$

Now, it is the weighting probability in the denominator that is not yet expressed in terms of observed data. This quantity does not appear to be recoverable. Indeed, we cannot re-express it in terms of observed variables since X is a parent of M_X and $\boldsymbol{Z_2}$ is a parent of $M_{\boldsymbol{Z_2}}$.

Recoverability of expectation of Y in m-DAGs E and I

For the expectation of Y in m-DAGs E and I, we show that both strategies also fail unless $M_Y \perp (M_{Z_2}, M_X) \mid (\boldsymbol{Z_1}, \boldsymbol{Z_2}, X)$.

- *Failure of g-formula-type identification*

$$E(Y^{\boldsymbol{M=0}}) = \sum P\left(Y^{\boldsymbol{M=0}} | \boldsymbol{Z_1}, X^{\boldsymbol{M=0}}, \boldsymbol{Z_2^{M=0}}\right) \times P\left(\boldsymbol{Z_1}, X^{\boldsymbol{M=0}}, \boldsymbol{Z_2^{M=0}}\right)$$

In both m-DAGs the second factor of the above expression is not recoverable because X and $\boldsymbol{Z_2}$ are parents of their missingness indicators (Theorem 3 of [9]) so it is not possible to move forward.

- *Failure of IPW-type identification unless* $M_Y \perp (M_{Z_2}, M_X) \mid (\boldsymbol{Z_1}, \boldsymbol{Z_2}, X)$

$$
\begin{aligned}
E(Y^{\boldsymbol{M=0}}) &= E\{E(Y^{\boldsymbol{M=0}}|X^{\boldsymbol{M=0}}, \boldsymbol{Z}_2^{\boldsymbol{M=0}}, \boldsymbol{Z_1})\} \\
&= E\left\{\frac{P(M_Y = 0|X^{\boldsymbol{M=0}}, \boldsymbol{Z}_2^{\boldsymbol{M=0}}, \boldsymbol{Z_1})}{P(M_Y = 0|X^{\boldsymbol{M=0}}, \boldsymbol{Z}_2^{\boldsymbol{M=0}}, \boldsymbol{Z_1})} E(Y^{\boldsymbol{M=0}}|X^{\boldsymbol{M=0}}, \boldsymbol{Z}_2^{\boldsymbol{M=0}}, \boldsymbol{Z_1})\right\} \\
&= E\left\{E\left(\frac{I(M_Y = 0)}{P(M_Y = 0|X^{\boldsymbol{M=0}}, \boldsymbol{Z}_2^{\boldsymbol{M=0}}, \boldsymbol{Z_1})}\middle|X^{\boldsymbol{M=0}}, \boldsymbol{Z}_2^{\boldsymbol{M=0}}, \boldsymbol{Z_1}\right) E(Y^{\boldsymbol{M=0}}|X^{\boldsymbol{M=0}}, \boldsymbol{Z}_2^{\boldsymbol{M=0}}, \boldsymbol{Z_1})\right\} \\
&= E\left\{E\left(\frac{I(M_Y = 0) \times Y^{\boldsymbol{M=0}}}{P(M_Y = 0|X^{\boldsymbol{M=0}}, \boldsymbol{Z}_2^{\boldsymbol{M=0}}, \boldsymbol{Z_1})}\middle|X^{\boldsymbol{M=0}}, \boldsymbol{Z}_2^{\boldsymbol{M=0}}, \boldsymbol{Z_1}\right)\right\} \\
&= E\left\{\frac{I(M_Y = 0) \times Y^{\boldsymbol{M=0}}}{P(M_Y = 0|X^{\boldsymbol{M=0}}, \boldsymbol{Z}_2^{\boldsymbol{M=0}}, \boldsymbol{Z_1})}\right\}.
\end{aligned}
$$

While the numerator in the last line becomes $I(M_Y = 0) \times Y$ by consistency, it is not possible to express the denominator in terms of observed data unless $M_Y \perp (M_{Z_2}, M_X) \mid (\boldsymbol{Z_1}, \boldsymbol{Z_2}, X)$. If this condition holds, then the expectation is recoverable as

$$
E(Y^{\boldsymbol{M=0}}) = E\left\{\frac{I(M_Y = 0) \times Y}{P(M_Y = 0|X, \boldsymbol{Z_2}, \boldsymbol{Z_1}, M_X = 0, M_{\boldsymbol{Z_2}} = 0)}\right\}.
$$

This entails the recoverability of the marginal distribution for Bernoulli-distributed Y but we were unable to establish the recoverability of the marginal outcome distribution in general.

Recoverability of expectation of X in m-DAG H

The proof is similar to that of the expectation of Y in m-DAGs E and I, but exchanging the roles of X and Y and considering the absence of an arrow from $\boldsymbol{Z_2}$ to $M_{\boldsymbol{Z_2}}$.

WEB APPENDIX 3: Simulation study

Overview

We performed a simulation study to investigate the performance of available case analysis and multiple imputation in conjunction with the proposed causal modelling approach. Our study closely mimicked the LSAC example but with a restricted number of confounders. Our data-generating mechanisms assumed main-effects models for the missingness indicators (with predictors according to the m-DAG) and set values of inestimable parameters, relating to the effect of a variable on its missingness indicator, to be of a similar magnitude to other associations observed in LSAC. We used approximately congenial imputation procedures for estimation. Specifically, we simulated 1000 complete data sets of size n=200 for each m-DAG as described next (simulation code can be downloaded at https://github.com/moreno-betancur/missingness_DAG).

Simulation models

We generated confounders, exposure and outcome according to the canonical c-DAG (Figure 1 of the main text). Specifically, we first drew a variable U from a standard normal distribution, and then we drew two binary confounders Z_1 and Z_2 from logistic regression models including U as predictor. A binary exposure X was then drawn from a logistic regression model including Z_1 and Z_2 as predictors, and a continuous outcome Y was drawn from a normal linear regression model including Z_1, Z_2 and X as predictors. Subsequently, we introduced missing data in each variable according to each of the ten canonical m-DAGs in turn, as well as for the "trivial" m-DAG with no arrows from c-DAG variables to missingness indicators. For this, we first drew a variable W from a standard normal distribution to represent an unmeasured common cause of the missingness indicators M_{Z_2}, M_X and M_Y. Then we drew M_{Z_2}, M_X and M_Y from three separate logistic regression models, each including W and the variables indicated by the corresponding m-DAG as predictors (main effects only).

Parameters of simulation models

We considered three scenarios for associations between substantive variables and missingness indicators: associations as in LSAC (Scenario 1), strong associations (Scenario 2) and very strong associations (Scenario 3). The parameter values used in the data generation models of Scenario 1 are given in Web Table 2. These values were determined by fitting equivalent models to the available data from LSAC wherever possible. We used wave 1 maternal completion of high school (Z_1), maternal smoking (Z_2) and maternal mental illness (X), and wave 3 SDQ score (Y), and their respective missingness indicators. Parameters related to U in generating the confounders were fixed so as to reflect the associations between the two counfounders and between these and the exposure in LSAC. Parameters related to W and intercept parameters were tuned so as to obtain missingness proportions similar to LSAC: around 23% for Y, 15% for X, and 15% for Z_2, and the proportion with any variable missing was 33-40% in each case. In tuning W parameters, we

also took care to preserve as far as possible the associations between missingness indicators observed in LSAC. Parameters relating to the effect of a variable on its missingness indicator were chosen to be of a magnitude similar to those observed for other associations in the data.

In Scenarios 2 and 3, we considered strong and very strong associations between substantive variables (Z_1, Z_2, X or Y) and missingness indicators relative to LSAC. We achieved this by doubling and tripling, respectively, the corresponding regression coefficients used to generate the missingness indicators in Scenario 1 (Web Table 2). The intercept parameters were in each case tuned to preserve similar proportions of missingness. The associations between W and the missingness indicators were not modified in these scenarios.

In secondary simulations, we investigated the role of W. Specifically, we repeated Scenario 1 but in the absence of W (i.e. such that missingness indicators are independent given the substantive variables). We also repeated Scenarios 2 and 3 but this time doubling or tripling the associations between W and the missingness indicators.

Analysis of simulated data sets

Estimates of the following target parameters were obtained in the complete data and in incomplete data sets for each missingness setting (determined by a combination of m-DAG and Scenario): the proportion exposed, the mean of Y and the coefficient of X in a linear regression of Y on X, Z_1 and Z_2. For missingness settings, we considered two approaches to handling missing data: the available case analysis and MICE with fifty imputations (based on relative efficiency assessments [10]) and five iterations (based on graphical convergence criteria [11]). For MICE, we used logistic imputation models for both Z_2 and X, and a linear imputation model for Y, with these models including all remaining variables as predictors (main effects only). Thus parametric models used were exact or close to those used to generate the data, and the procedure followed current recommendations for congenial imputations.

Indicators of performance of methods

We calculated the mean, relative bias, empirical standard error and standardized bias of estimates obtained with each approach for each m-DAG and Scenario, using the mean of the complete data estimates as benchmark for bias estimation. The standardized bias indicates the impact of bias in terms of conclusions from tests and confidence intervals given the variability [12].

Results

Recoverable parameters

For recoverable parameters, across all three scenarios, the available case analysis exhibited small standardized biases in the expected settings, i.e. trivial m-DAG (all parameters) and m-DAGs A, B, D and E (regression coefficient) (see Web Figure 1 in the main text; detailed results are shown in Web Tables 3–5). This approach is expected to be subject to selection

bias for all other recoverable parameters. In these simulations, the bias was non-negligible for the outcome mean: across the three scenarios in m-DAGs B and C; in Scenarios 2 and 3 for m-DAGs A and D; and in Scenario 3 for m-DAG F. The bias was non-negligible in Scenario 3 for the proportion exposed and the regression coefficient in m-DAG D. MICE had small standardized biases for all recoverable parameters, showing that misspecification bias was not an issue in these simulations. A gain in precision of MICE over the available case analysis was noticeable for the regression coefficient.

Non-recoverable parameters

For non-recoverable mean parameters, both methods exhibited non-negligible standardized biases with the exception of a few cases. Biases became larger with stronger associations (Scenarios 2 and 3) and were larger with available case analysis than with MICE. When it was non-recoverable, the regression coefficient was estimated with negligible bias by both approaches in Scenario 1, and while the size of biases in both methods was larger in Scenarios 2 and 3, it remained negligible except for m-DAG H in Scenario 3 with both approaches.

Secondary simulations

In our secondary simulations (results not shown), we found that absence of W in Scenario 1 led to biases of larger magnitude where they existed. Conversely, when strengthening the associations between W and missingness indicators in Scenarios 2 and 3, biases were of smaller magnitude. These findings suggest that correlations between missingness indicators that arise from unmeasured factors that are unrelated to the substantive variables are beneficial in terms of bias magnitudes.

WEB TABLES

Web Table 1. Conditional independence properties implied by the m-DAGs of Figure 2 in the main text

m-DAG	Conditional Independence Properties
A	$(M_{Z_2}, M_X, M_Y) \perp (\boldsymbol{Z_2}, X, Y) \mid \boldsymbol{Z_1}$
B	$M_{Z_2} \perp (\boldsymbol{Z_2}, Y) \mid (\boldsymbol{Z_1}, X)$<br>$M_X \perp (X, Y) \mid (\boldsymbol{Z_1}, \boldsymbol{Z_2})$<br>$M_Y \perp Y \mid (\boldsymbol{Z_1}, \boldsymbol{Z_2}, X)$
C	$M_{Z_2} \perp \boldsymbol{Z_2} \mid (\boldsymbol{Z_1}, X, Y)$<br>$M_X \perp X \mid (\boldsymbol{Z_1}, \boldsymbol{Z_2}, Y)$<br>$M_Y \perp Y \mid (\boldsymbol{Z_1}, \boldsymbol{Z_2}, X)$
D	$M_{Z_2} \perp (X, Y) \mid (\boldsymbol{Z_1}, \boldsymbol{Z_2})$<br>$M_X \perp (\boldsymbol{Z_2}, Y) \mid (\boldsymbol{Z_1}, X)$<br>$M_Y \perp (\boldsymbol{Z_2}, X, Y) \mid \boldsymbol{Z_1}$
E	$(M_{Z_2}, M_X, M_Y) \perp Y \mid (\boldsymbol{Z_1}, \boldsymbol{Z_2}, X)$
F	$M_{Z_2} \perp X \mid (Y, \boldsymbol{Z_1}, \boldsymbol{Z_2})$<br>$M_X \perp \boldsymbol{Z_2} \mid (X, Y, \boldsymbol{Z_1})$<br>$M_Y \perp (\boldsymbol{Z_2}, X, Y) \mid \boldsymbol{Z_1}$
G	$M_{Z_2} \perp (\boldsymbol{Z_2}, Y) \mid (\boldsymbol{Z_1}, X)$<br>$M_X \perp (X, Y) \mid (\boldsymbol{Z_1}, \boldsymbol{Z_2})$
H	$M_{Z_2} \perp \boldsymbol{Z_2} \mid (\boldsymbol{Z_1}, X, Y)$<br>$M_X \perp X \mid (\boldsymbol{Z_1}, \boldsymbol{Z_2}, Y)$
I	$M_Y \perp Y \mid (\boldsymbol{Z_1}, \boldsymbol{Z_2}, X)$
J	None

Web Table 2. Regression coefficients of data generation models used to simulate each variable in Scenario 1 of the simulation study

	**Model for:**	**Regression coefficient of:**						
		**Intercept**	$\boldsymbol{Z_1}$	$\boldsymbol{Z_2}$	$\boldsymbol{X}$	$\boldsymbol{Y}$	$\boldsymbol{U}$	$\boldsymbol{W}$
All DAGs	Z_1	0.43					1.40	
	Z_2	-1.66					-1.40	
	X	-1.35	-0.20	0.50				
	Y [a]	7.36	-1.23	1.42	2.60			
Trivial m-DAG	M_{Z_2}	-4.34						4.00
	M_X	-5.26						5.00
	M_Y	-1.36						0.90
m-DAG A	M_{Z_2}	-2.40	-0.45					2.00
	M_X	-3.17	-0.45					3.00
	M_Y	-0.99	-0.63					0.80
m-DAG B	M_{Z_2}	-2.20	-0.45		0.50			1.50
	M_X	-2.90	-0.45	0.50				2.50
	M_Y	-1.30	-0.46	0.50	0.47			0.80
m-DAG C	M_{Z_2}	-2.30	-0.25		0.50	0.03		1.20
	M_X	-2.90	-0.25	0.50		0.03		2.20
	M_Y	-1.30	-0.46	0.50	0.47			0.80
m-DAG D	M_{Z_2}	-2.00	-0.45	0.50				1.50
	M_X	-3.00	-0.45		0.50			2.50
	M_Y	-0.99	-0.63					0.80
m-DAG E	M_{Z_2}	-2.20	-0.45	0.50	0.50			1.50
	M_X	-2.90	-0.45	0.50	0.50			2.50
	M_Y	-1.30	-0.46	0.50	0.47			0.80
m-DAG F	M_{Z_2}	-3.00	-0.25	0.50		0.03		2.00
	M_X	-4.00	-0.25		0.50	0.03		3.30
	M_Y	-1.70	-0.63					2.00
m-DAG G	M_{Z_2}	-2.20	-0.45	0.50	0.50			1.50
	M_X	-2.90	-0.45	0.50	0.50			2.50
	M_Y	-3.00	-0.46	0.50	0.47	0.03		3.00
m-DAG H	M_{Z_2}	-2.60	-0.25	0.50	0.50	0.03		1.50
	M_X	-3.50	-0.25	0.50	0.50	0.03		2.50
	M_Y	-2.20	-0.46	0.50	0.47	0.03		2.00
m-DAG I	M_{Z_2}	-2.60	-0.25	0.50	0.50	0.03		1.50
	M_X	-3.20	-0.25	0.50	0.50	0.03		2.50
	M_Y	-1.30	-0.46	0.50	0.47			0.80
m-DAG J	M_{Z_2}	-2.60	-0.45	0.50				2.00
	M_X	-3.50	-0.45		0.50			3.00
	M_Y	-1.20	-0.63			0.03		0.80

[a] The residual error standard deviation was 5.04.

Web Table 3. Simulation study results for Scenario 1 (associations as in LSAC): performance indicators [a] for two approaches for dealing with missing data across the m-DAGs of Figure 2 of the main text, as well as for the trivial m-DAG [b]

m-DAG	Strategy	Proportion exposed, $E(X)$				Mean of outcome, $E(Y)$				Fully adjusted mean difference [c]			
		Mean	RelBias %	ESE	StdBias %	Mean	RelBias %	ESE	StdBias %	Mean	RelBias %	ESE	StdBias %
c-DAG	Complete data [d]	0.21	0	0.03	0	7.51	0	0.35	0	2.60	0	0.93	0
Trivial	Available cases	0.21	0.2	0.03	1.6	7.49	-0.2	0.41	-3.0	2.59	-0.3	1.15	-0.6
	MICE	0.21	1.3	0.03	8.7	7.50	-0.1	0.42	-2.1	2.60	0.2	1.10	0.5
A	Available cases	0.21	-0.5	0.03	-3.0	7.46	-0.7	0.41	-11.9	2.62	1.0	1.17	2.3
	MICE	0.21	0.9	0.03	5.9	7.51	0.1	0.41	1.8	2.61	0.6	1.10	1.3
B	Available cases	0.21	-0.7	0.03	-4.6	7.38	-1.7	0.41	-31.4	2.58	-0.5	1.22	-1.0
	MICE	0.21	0.8	0.03	5.6	7.49	-0.2	0.41	-3.1	2.57	-0.9	1.16	-2.1
C	Available cases	0.21	-1.3	0.03	-8.7	7.38	-1.6	0.42	-29.5	2.55	-1.6	1.28	-3.3
	MICE	0.21	0.8	0.03	5.7	7.49	-0.2	0.42	-3.7	2.56	-1.2	1.16	-2.7
D	Available cases	0.20	-3.8	0.03	-25.7	7.44	-0.9	0.42	-16.7	2.54	-2.3	1.25	-4.7
	MICE	0.20	-2.5	0.03	-16.8	7.49	-0.2	0.41	-4.5	2.55	-1.9	1.15	-4.3
E	Available cases	0.20	-5.2	0.03	-35.9	7.37	-1.7	0.41	-32.0	2.60	0.1	1.31	0.2
	MICE	0.20	-3.4	0.03	-23.8	7.48	-0.3	0.41	-6.3	2.58	-0.5	1.21	-1.1
F	Available cases	0.20	-3.9	0.03	-26.2	7.47	-0.5	0.41	-8.6	2.58	-0.7	1.16	-1.7
	MICE	0.20	-2.4	0.03	-16.4	7.50	-0.1	0.41	-1.7	2.59	-0.4	1.11	-1.0
G	Available cases	0.21	-1.0	0.03	-6.5	7.36	-1.9	0.40	-36.1	2.55	-1.6	1.18	-3.5
	MICE	0.21	0.5	0.03	3.4	7.42	-1.1	0.40	-20.2	2.55	-1.9	1.11	-4.5
H	Available cases	0.21	-1.3	0.03	-8.8	7.29	-2.9	0.41	-52.9	2.55	-1.7	1.16	-3.7
	MICE	0.21	0.2	0.03	1.7	7.37	-1.7	0.41	-31.8	2.55	-1.7	1.10	-4.1
I	Available cases	0.20	-5.5	0.03	-36.7	7.39	-1.6	0.41	-28.5	2.61	0.4	1.28	0.8
	MICE	0.20	-3.5	0.03	-23.2	7.49	-0.2	0.41	-3.5	2.61	0.6	1.21	1.2
J	Available cases	0.20	-3.4	0.03	-23.1	7.28	-3.0	0.40	-56.3	2.56	-1.2	1.18	-2.7
	MICE	0.20	-2.3	0.03	-15.5	7.34	-2.2	0.39	-41.6	2.58	-0.4	1.15	-1.0

[a] Relative Bias (RelBias)= 100*(Mean/Benchmark - 1); Standardized Bias (StdBias)= 100*(Mean-Benchmark)/ESE; ESE – Empirical standard error

[b] The shaded area indicates non-recoverable parameters.

[c] Defined as the coefficient of X in a linear regression of Y on X, Z_1, Z_2.

[d] Benchmark for bias estimations.

Web Table 4. Simulation study results for Scenario 2 (strong associations between substantive variables and missingness indicators): performance indicators [a] for two approaches for dealing with missing data across the m-DAGs of Figure 2 of the main text, as well as for the trivial m-DAG [b]

m-DAG	Strategy	Proportion exposed, $E(X)$				Mean of outcome, $E(Y)$				Fully adjusted mean difference [c]			
		Mean	RelBias %	ESE	StdBias %	Mean	RelBias %	ESE	StdBias %	Mean	RelBias %	ESE	StdBias %
c-DAG	Complete data [d]	0.21	0	0.03	0	7.50	0	0.38	0	2.63	0	0.86	0
Trivial	Available cases	0.21	-0.1	0.03	-0.7	7.51	0.1	0.43	2.4	2.62	-0.2	1.09	-0.5
	MICE	0.21	1.0	0.03	6.6	7.51	0.2	0.43	2.6	2.62	-0.6	1.06	-1.4
A	Available cases	0.21	-0.5	0.03	-3.1	7.40	-1.2	0.43	-21.6	2.58	-2.0	1.07	-4.8
	MICE	0.21	1.0	0.03	6.8	7.49	-0.1	0.43	-1.0	2.58	-1.9	1.05	-4.8
B	Available cases	0.20	-1.3	0.03	-8.5	7.25	-3.3	0.42	-57.6	2.65	0.7	1.20	1.6
	MICE	0.21	0.8	0.03	5.4	7.48	-0.2	0.44	-4.0	2.59	-1.6	1.12	-3.7
C	Available cases	0.20	-2.9	0.03	-18.9	7.22	-3.7	0.43	-63.3	2.43	-7.8	1.41	-14.6
	MICE	0.21	0.6	0.03	4.1	7.44	-0.8	0.45	-12.7	2.59	-1.4	1.20	-3.0
D	Available cases	0.19	-7.9	0.03	-55.4	7.41	-1.1	0.42	-20.3	2.63	0.0	1.14	0.0
	MICE	0.19	-6.2	0.03	-43.7	7.49	0.0	0.42	-0.2	2.61	-0.6	1.08	-1.6
E	Available cases	0.19	-10.1	0.03	-69.3	7.24	-3.4	0.41	-61.4	2.63	0.1	1.33	0.2
	MICE	0.19	-7.9	0.03	-54.6	7.44	-0.8	0.43	-13.6	2.60	-1.0	1.24	-2.2
F	Available cases	0.19	-8.2	0.03	-58.8	7.45	-0.7	0.41	-12.6	2.62	-0.3	1.08	-0.7
	MICE	0.20	-6.0	0.03	-42.2	7.49	0.0	0.41	-0.8	2.62	-0.3	1.03	-0.7
G	Available cases	0.20	-1.3	0.03	-8.5	7.18	-4.2	0.41	-77.1	2.56	-2.8	1.17	-6.3
	MICE	0.21	0.7	0.03	4.5	7.30	-2.5	0.42	-45.9	2.52	-4.2	1.10	-10.0
H	Available cases	0.20	-2.3	0.03	-15.2	7.06	-5.9	0.43	-102.4	2.47	-6.3	1.19	-13.8
	MICE	0.21	0.0	0.03	0.1	7.22	-3.6	0.43	-63.2	2.50	-5.0	1.09	-12.1
I	Available cases	0.18	-13.2	0.03	-92.6	7.24	-3.4	0.43	-58.3	2.53	-3.9	1.37	-7.6
	MICE	0.19	-9.8	0.03	-67.7	7.43	-0.9	0.44	-16.0	2.60	-1.1	1.20	-2.5
J	Available cases	0.19	-6.7	0.03	-46.4	7.08	-5.6	0.43	-98.5	2.65	0.7	1.17	1.6
	MICE	0.20	-5.5	0.03	-38.0	7.17	-4.3	0.43	-75.6	2.65	0.8	1.11	1.8

[a] Relative Bias (RelBias)= 100*(Mean/Benchmark - 1); Standardized Bias (StdBias)= 100*(Mean-Benchmark)/ESE; ESE – Empirical standard error
[b] The shaded area indicates non-recoverable parameters.
[c] Defined as the coefficient of X in a linear regression of Y on X, Z_1, Z_2.
[d] Benchmark for bias estimations.

Web Table 5. Simulation study results for Scenario 3 (very strong associations between substantive variables and missingness indicators): performance indicators [a] for two approaches for dealing with missing data across the m-DAGs of Figure 2 of the main text, as well as for the trivial m-DAG [b]

m-DAG	Strategy	Proportion exposed, $E(X)$				Mean of outcome, $E(Y)$				Fully adjusted mean difference [c]			
		Mean	RelBias %	ESE	StdBias %	Mean	RelBias %	ESE	StdBias %	Mean	RelBias %		ESE %
c-DAG	Complete data [d]	0.21	0	0.03	0	7.50	0	0.37	0	2.60	0	0.90	0
Trivial	Available cases	0.21	0.2	0.03	1.2	7.51	0.0	0.43	0.3	2.57	-1.0	1.14	-2.4
	MICE	0.21	1.4	0.03	9.5	7.51	0.1	0.42	0.9	2.58	-0.8	1.10	-2.0
A	Available cases	0.21	-0.4	0.03	-2.7	7.39	-1.5	0.41	-27.7	2.62	0.7	1.13	1.6
	MICE	0.21	1.0	0.03	6.9	7.50	-0.1	0.41	-1.7	2.61	0.3	1.08	0.8
B	Available cases	0.20	-2.0	0.03	-13.5	7.14	-4.8	0.43	-83.5	2.53	-2.8	1.35	-5.3
	MICE	0.21	0.6	0.03	3.9	7.46	-0.6	0.46	-10.1	2.48	-4.6	1.26	-9.5
C	Available cases	0.20	-5.0	0.03	-33.0	7.14	-4.9	0.42	-87.4	2.20	-15.5	1.56	-25.9
	MICE	0.21	0.1	0.03	0.6	7.44	-0.9	0.44	-15.8	2.55	-1.8	1.29	-3.7
D	Available cases	0.18	-11.7	0.03	-82.4	7.40	-1.4	0.41	-25.5	2.61	0.3	1.18	0.7
	MICE	0.19	-10.1	0.03	-71.0	7.50	-0.1	0.42	-1.7	2.58	-0.6	1.12	-1.5
E	Available cases	0.18	-15.3	0.03	-105.6	7.15	-4.7	0.43	-82.5	2.61	0.3	1.42	0.5
	MICE	0.18	-13.1	0.03	-89.6	7.41	-1.2	0.45	-20.2	2.59	-0.2	1.31	-0.5
F	Available cases	0.18	-13.8	0.03	-96.4	7.43	-1.0	0.40	-19.0	2.48	-4.7	1.19	-10.2
	MICE	0.18	-10.7	0.03	-73.5	7.49	-0.2	0.40	-2.9	2.52	-3.2	1.12	-7.5
G	Available cases	0.20	-2.1	0.03	-14.1	7.01	-6.6	0.41	-120.6	2.50	-3.9	1.30	-7.8
	MICE	0.21	0.4	0.03	2.4	7.20	-4.1	0.42	-72.4	2.40	-7.7	1.19	-16.6
H	Available cases	0.20	-4.4	0.03	-29.1	6.82	-9.1	0.43	-159.7	2.14	-17.6	1.39	-33.1
	MICE	0.20	-1.1	0.03	-7.2	7.05	-6.0	0.45	-101.4	2.28	-12.4	1.21	-26.6
I	Available cases	0.16	-21.7	0.03	-148.8	7.15	-4.8	0.41	-86.4	2.33	-10.6	1.58	-17.4
	MICE	0.17	-17.3	0.03	-116.7	7.38	-1.7	0.43	-29.4	2.59	-0.5	1.38	-0.9
J	Available cases	0.19	-9.8	0.03	-70.1	6.92	-7.8	0.42	-138.5	2.57	-1.1	1.16	-2.5
	MICE	0.19	-8.6	0.03	-61.6	7.03	-6.4	0.43	-110.6	2.57	-1.2	1.11	-2.8

[a] Relative Bias (RelBias)= 100*(Mean/Benchmark - 1); Standardized Bias (StdBias)= 100*(Mean-Benchmark)/ESE; ESE – Empirical standard error

[b] The shaded area indicates non-recoverable parameters.

[c] Defined as the coefficient of X in a linear regression of Y on X, Z_1, Z_2.

[d] Benchmark for bias estimations.

WEB FIGURES

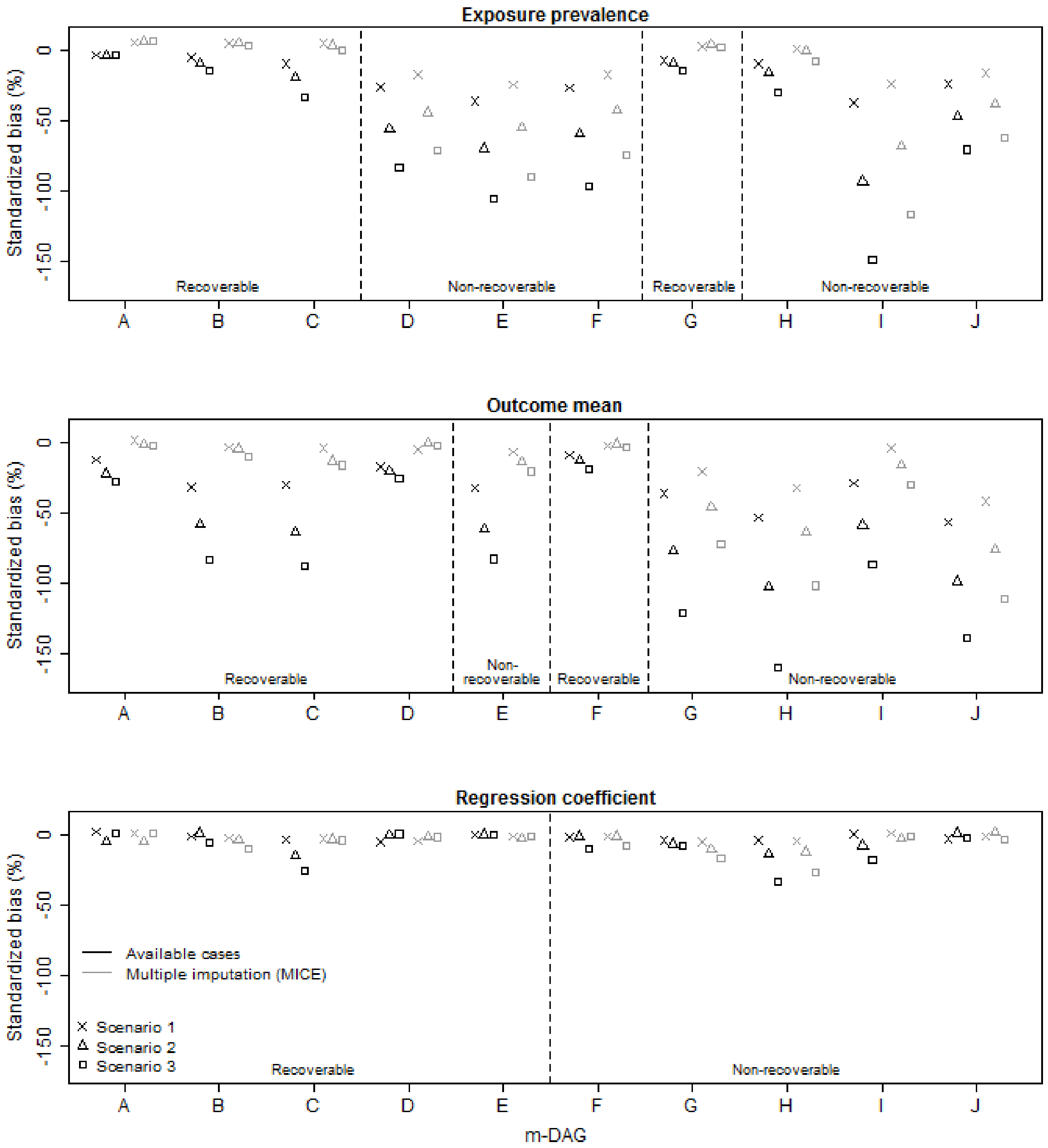

Web Figure 1. Simulation study results: Standardized bias of available case and multiple imputation by chained equations (MICE) approaches when estimating three parameters in each canonical m-DAG across three scenarios for associations between missingness indicators and substantive variables: Scenario 1 (LSAC-type associations), Scenario 2 (strong associations) and Scenario 3 (very strong associations).

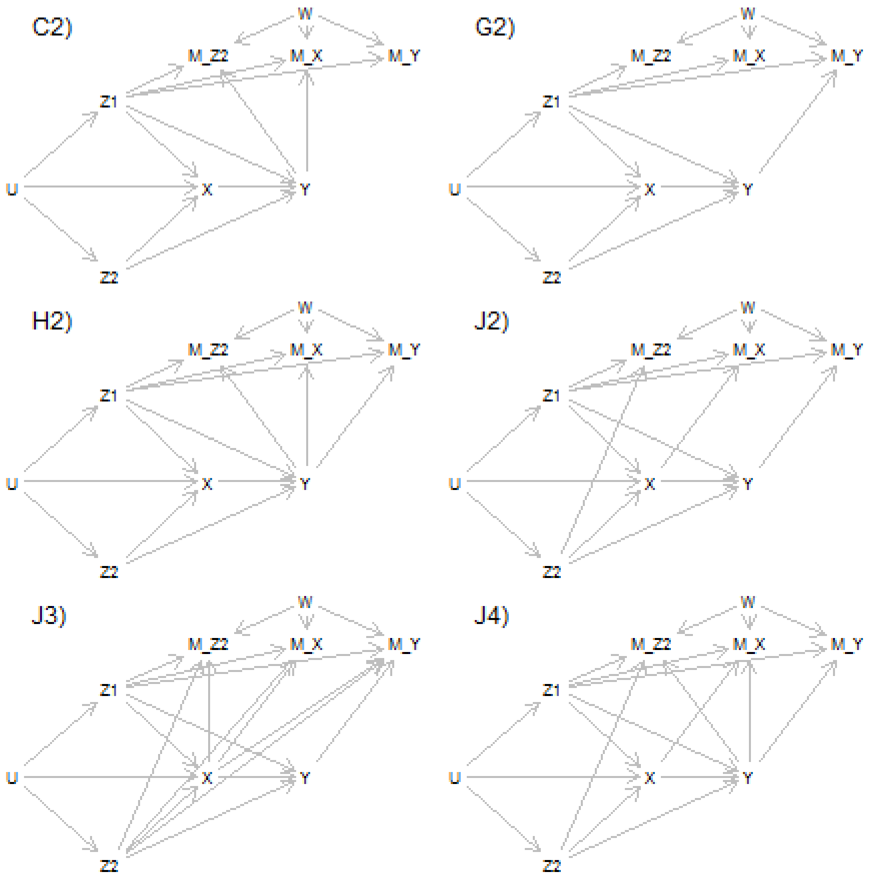

Web Figure 2. The remaining six canonical m-DAGs that were not explicitly considered in the main text.

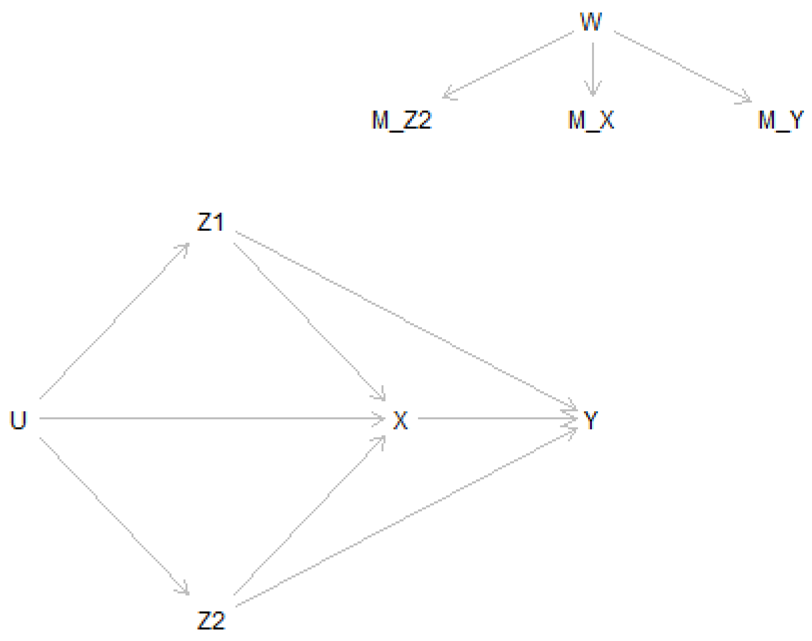

Web Figure 3. The "trivial" m-DAG with no arrows from c-DAG variables to missingness indicators (corresponds to the "missing completely at random" assumption).

REFERENCES

1. Textor J, van der Zander B. dagitty: Graphical Analysis of Structural Causal Models [Internet]. 2016. Available from: https://cran.r-project.org/package=dagitty
2. Mohan K, Pearl J, Tian J. Graphical Models for Inference with Missing Data. In: Burges C, Bottou L, Welling M, Ghahramani Z, Weinberger K, editors. Advances in Neural Information Processing Systems 26 (NIPS 2013). Red Hook, NY: Curran Associates, Inc; 2013. p. 1277–85.
3. Hernan MA, Robins JM. Causal Inference. Boca Raton, FL: Chapman and Hall/CRC, forthcoming; 2018.
4. Pearl J. Causality: models, reasoning, and inference. New York, New York: Cambridge University Press; 2000.
5. Mohan K, Pearl J. On the testability of models with missing data. Proc AISTAT-2014. 2014;(April):643–50.
6. Shpitser I, Mohan K, Pearl J. Missing Data as a Causal and Probabilistic Problem. Proc Thirty-First Conf Uncertain Artif Intell. 2015;(July):802–11.
7. Mohan K, Pearl J. Graphical Models for Processing Missing Data [Internet]. 2018. Available from: http://arxiv.org/abs/1801.03583 (accessed April 1 2018)
8. Shpitser I, Robins JM. Towards A Complete Identification Algorithm for Missing Data Problems. In: What If? Inference and Learning of Hypothetical and Counterfactual Interventions in Complex Systems: Workshop at NIPS 2016 conference. 2016. p. 1–3.
9. Mohan K, Pearl J. Graphical Models for Recovering Probabilistic and Causal Queries from Missing Data. In: Ghahramani Z, Welling M, Cortes C, Lawrence N, Weinberger K, editors. Advances in Neural Information Processing Systems 27 (NIPS 2014). Red Hook, NY: Curran Associates, Inc; 2014. p. 1520–8.
10. Rubin DB. Multiple Imputation for Nonresponse in Surveys. New York, New York: Wiley; 1987.
11. van Buuren S, Groothuis-Oudshoorn K. mice: Multivariate Imputation by Chained Equations in R. J Stat Softw. 2011;45(3):1–67.
12. Collins LM, Schafer JL, Kam C. A Comparison of Inclusive and Restrictive Strategies in Modern Missing Data Procedures. Psychol Methods. 2001;6(4):330–51.
