# Supplementary material for: Correction to: “Canonical causal diagrams to guide the treatment of missing data in epidemiologic studies”
Source: Am J Epidemiol. 2025 Jan 17;194(3):877–80. doi: 10.1093/aje/kwae406 (PMC11879509; doi:10.1093/aje/kwae406)
Supplement: Web_Material_kwae406 [file web_material_kwae406.zip › Moreno-Betancur_Supplementary material_11oct2024.pdf]

Supplementary Material

Correction to "Canonical Causal Diagrams to Guide the Treatment of Missing Data in Epidemiologic Studies"[1]

Margarita Moreno-Betancur, Katherine J. Lee, Finbarr P. Leacy,
Julie A. Simpson, John B. Carlin

TABLE OF CONTENTS

- **Appendix S1: Proofs for corrected recoverability results**
  - m-DAG C (all distributions)
  - m-DAG B (joint and marginal distributions)
  - m-DAG G (marginal exposure distribution)

- **Appendix S2: Proofs for results that are newly established following the correction**
  - m-DAG E (marginal outcome distribution)
  - m-DAG H (marginal exposure distribution)
  - m-DAG H2 (marginal exposure distribution)
  - m-DAG I (marginal and conditional outcome distribution)
  - m-DAG F (conditional outcome distribution)

- **Appendix S3: Detailed proofs for unchanged results**
  - m-DAG C2 (all distributions)
  - m-DAG G2 (all distributions)

- **References for Appendix S1-S3**

- **Figures and tables for Appendix S1-S3**
  - Figure S1: Reduced versions of canonical m-DAGs C, B, F and H2 used in the proofs in Appendix S1-S2
  - Table S1: Two models with distributions compatible with m-DAG F ' in Figure S1
  - Table S2: Observed distribution with missing data common to models $\mathcal{M}_1$ and $\mathcal{M}_2$

APPENDIX S1 - PROOFS FOR CORRECTED RECOVERABILITY RESULTS

This appendix provides proofs for the corrected recoverability results. Notation and assumptions are as in the original article.

Non-recoverability of all target distributions in m-DAG C

By Mohan,[2] if a parameter is not recoverable in m-DAG C' in Figure S1 in this document, then it is not recoverable in m-DAG C, which is obtained by adding edges to m-DAG C'.

To prove non-recoverability in m-DAG C' for each of the target distributions, we draw on results by Nabi and colleagues[3,4] who prove non-recoverability of some parameters in an m-DAG with a so-called criss-cross structure.

Considering the extreme scenario where $W = M_X$, it is seen that m-DAG C' reduces to the criss-cross structure shown in m-DAG C" in Figure S1. By the counterexamples provided by Nabi and colleagues[3,4], in this m-DAG neither the marginal or conditional outcome distributions are recoverable, nor is the marginal exposure distribution. This can be seen for example by computing each distribution in each of the two models provided in Nabi et al.[3] and confirming they are not equal. This implies that the joint distribution is not recoverable in m-DAG C" either. This counterexample proves that none of the four parameters of interest is recoverable in m-DAG C' nor, as result, m-DAG C.

Non-recoverability of joint and marginal distributions in m-DAG B

Consider m-DAG B' in Figure S1 of this document, obtained by removing edges from m-DAG B. By Mohan,[2] if a parameter is not recoverable in m-DAG B', then it is not recoverable in m-DAG B. Substituting Z_2 (assumed univariate, binary) for X, and X for Y in the proof for m-DAG C provided above establishes that the marginal distribution of X is not recoverable in m-DAG B' and thus neither is the joint distribution.

To prove non-recoverability of the marginal distribution of Y in m-DAG B, we consider m-DAG B", which is also obtained by removing edges from m-DAG B, and more specifically m-DAG B"', which collapses X and $\boldsymbol{Z_2}$ into a single node $\boldsymbol{V}$, where $M_{\boldsymbol{V}} = 1$ if either $M_{\boldsymbol{Z_2}} = 1$ or $M_X = 1$, and $M_{\boldsymbol{V}} = 0$ otherwise. The simplification in m-DAG B"' retains the detail necessary for examining the recoverability of $P(Y^{\boldsymbol{M}=\boldsymbol{0}} = y)$, because separating Y from M_Y requires conditioning on both X and $\boldsymbol{Z_2}$ in this m-DAG, which means it is contingent on the recoverability of $P(\boldsymbol{V}^{\boldsymbol{M}=\boldsymbol{0}} = \boldsymbol{v})$. Indeed:

$$\begin{aligned}
P(Y^{\boldsymbol{M}=\boldsymbol{0}} = y) &= \sum_{x,\boldsymbol{z_2}} P(Y^{\boldsymbol{M}=\boldsymbol{0}} = y \mid X^{\boldsymbol{M}=\boldsymbol{0}} = x, \boldsymbol{Z}_{\boldsymbol{2}}^{\boldsymbol{M}=\boldsymbol{0}} = \boldsymbol{z_2}) \times P(X^{\boldsymbol{M}=\boldsymbol{0}} = x, \boldsymbol{Z}_{\boldsymbol{2}}^{\boldsymbol{M}=\boldsymbol{0}} = \boldsymbol{z_2}) \\
&= \sum_{x,\boldsymbol{z_2}} P(Y = y \mid X = x, \boldsymbol{Z_2} = \boldsymbol{z_2}, M_Y = 0, M_X = 0, M_{\boldsymbol{Z_2}} = 0) \times P(X^{\boldsymbol{M}=\boldsymbol{0}} = x, \boldsymbol{Z}_{\boldsymbol{2}}^{\boldsymbol{M}=\boldsymbol{0}} = \boldsymbol{z_2}) \\
&= \sum_{x,\boldsymbol{z_2}} P(Y = y \mid \boldsymbol{V} = \boldsymbol{v}, M_Y = 0, M_{\boldsymbol{V}} = 0) \times P(\boldsymbol{V}^{\boldsymbol{M}=\boldsymbol{0}} = \boldsymbol{v})
\end{aligned}$$

The first term of this sum is expressed in terms of observable data, and so is recoverable, but the second term, i.e. $P(\boldsymbol{V}^{\boldsymbol{M}=\boldsymbol{0}} = \boldsymbol{v})$, is not, given that the marginal distribution of X is not recoverable, per above. Using this expression, the non-recoverability of the marginal outcome distribution follows per the proof in Appendix A6 of Zhang et al.[5]

Non-recoverability of the marginal distribution of X in m-DAG G

By Mohan,[2] the non-recoverability of the marginal distribution of X in m-DAG G follows from the non-recoverability of this parameter in m-DAG B, given that G is obtained by adding a single arrow to B.

APPENDIX S2 - PROOFS FOR RESULTS THAT ARE NEWLY ESTABLISHED FOLLOWING THE CORRECTION

This appendix provides proofs for results that had been left as open questions in the original manuscript and are now established as a result of the correction. Notation and assumptions are as in the original article.

Non-recoverability of the marginal distribution of Y in m-DAG E

By Mohan,[2] the non-recoverability of the marginal distribution of Y in m-DAG E follows from the non-recoverability of this distribution in m-DAG B, given that E is obtained by adding arrows to B.

Non-recoverability of the marginal distribution of X in m-DAG H

By Mohan,[2] the non-recoverability of the marginal distribution of X in m-DAG H follows from the non-recoverability of this distribution in m-DAG G, given that H is obtained by adding arrows to G.

Non-recoverability of the marginal distribution of X in m-DAG H2

The proof follows similarly as that for the non-recoverability of the marginal distribution of Y in m-DAG B'''. To prove non-recoverability of the marginal distribution of X in m-DAG H2, we consider m-DAG H2', which is obtained by removing edges from m-DAG H2. Separating X from M_X requires conditioning on both Y in this m-DAG, which means it is contingent on the recoverability of $P(Y^{\boldsymbol{M=0}} = y)$. Indeed:

$$\begin{aligned} P(X^{\boldsymbol{M=0}} = x) \quad &= \sum_y P(X^{\boldsymbol{M=0}} = x | Y^{\boldsymbol{M=0}} = y) \times P(Y^{\boldsymbol{M=0}} = y) \\ &= \sum_y P(X = x | Y = y, M_Y = 0, M_X = 0) \times P(Y^{\boldsymbol{M=0}} = y) \end{aligned}$$

The first term of this sum is expressed in terms of observable data, and so is recoverable, but the second term, i.e. $P(Y^{\boldsymbol{M=0}} = y)$, is not recoverable, by Theorem 3 of Mohan and Pearl [6] because Y is a cause of M_Y. Using this expression, the non-recoverability of the marginal exposure distribution follows per the proof in Appendix A6 of Zhang et al.[5]

Non-recoverability of the marginal and conditional distributions of Y in m-DAG I

By Mohan,[2] the non-recoverability of the marginal distribution of Y in m-DAG I follows from the non-recoverability of this distribution in m-DAG B, given that I is obtained by adding arrows to B. Similarly, the non-recoverability of the conditional distribution of Y in m-DAG I follows from the non-recoverability of this distribution in m-DAG C, given that I is obtained by adding arrows to C.

Non-recoverability of the conditional distribution of Y in m-DAG F

By Mohan,[2] if a parameter is not recoverable in m-DAG F' in Figure S1 in this document, then it is not recoverable in m-DAG F, which is obtained by adding edges to m-DAG F'.

To prove non-recoverability of the conditional distribution of Y in m-DAG F' we provide a counterexample to recoverability of this distribution considering the case where X, Y, M_X and M_Y are binary, and $W = M_X$, as in m-DAG F'' in Figure S1. Specifically, in Table S1 we provide two models, $\mathcal{M}_1$ and $\mathcal{M}_2$, that are compatible with m-DAG F'' and that agree on observed distributions but disagree on the conditional distribution of Y, as we now show.

Claim 1: The models in Table S1 are compatible with m-DAG F''

The conditional independencies implied by the m-DAG hold in the two models:

- $X \perp M_Y$

  Model $\mathcal{M}_1$:
  $$P(M_Y = 1|X = 1) = P(M_Y = 1|X = 0) = \frac{1}{2}$$

  Model $\mathcal{M}_2$:
  $$P(M_Y = 1|X = 1) = P(M_Y = 1|X = 0) = \frac{1}{2}$$

- $Y \perp M_Y$

  Model $\mathcal{M}_1$:
  $$P(M_Y = 1|Y = 1) = P(M_Y = 1|Y = 0) = \frac{1}{2}$$

  Model $\mathcal{M}_2$:
  $$P(M_Y = 1|Y = 1) = P(M_Y = 1|Y = 0) = \frac{1}{2}$$

Claim 2: The models in Table S1 agree on the observed distributions

The distribution that would be observed with missing data is identical for both models. This distribution is shown in Table S2.

In particular, it can be confirmed that:

- For all x, y, $P^{\mathcal{M}_1}(x, y, 0,0) = P^{\mathcal{M}_2}(x, y, 0,0)$ as the top four rows of Table S1 for model $\mathcal{M}_1$ are identical to the top four rows for model $\mathcal{M}_2$ (and equal to the top four rows of Table S2).
- $P^{\mathcal{M}_1}(X = 1, M_X = 0) = P^{\mathcal{M}_2}(X = 1, M_X = 0) = \frac{14}{32}$
- $P^{\mathcal{M}_1}(X = 0, M_X = 0) = P^{\mathcal{M}_2}(X = 0, M_X = 0) = \frac{1}{16}$
- $P^{\mathcal{M}_1}(Y = 1, M_Y = 0) = P^{\mathcal{M}_2}(Y = 1, M_Y = 0) = \frac{9}{32}$
- $P^{\mathcal{M}_1}(Y = 0, M_Y = 0) = P^{\mathcal{M}_2}(Y = 0, M_Y = 0) = \frac{7}{32}$

Claim 3: The models in Table S1 disagree on the conditional outcome distribution

$$P^{\mathcal{M}_1}(Y = 1|X = 0, \boldsymbol{Z_1}, \boldsymbol{Z_2}) = P^{\mathcal{M}_1}(Y = 1|X = 0) = \frac{6}{10} \neq \frac{2}{6} = P^{\mathcal{M}_2}(Y = 1|X = 0) = P^{\mathcal{M}_2}(Y = 1|X = 0, \boldsymbol{Z_1}, \boldsymbol{Z_2})$$

APPENDIX S3 - DETAILED PROOFS FOR UNCHANGED RESULTS

This appendix provides detailed proofs for results that have not changed, but for which detailed proofs were not provided previously as the results were claimed to follow from some of the incorrect results. Notation and assumptions are as in the original article.

Recoverability results for m-DAG C2

Next, we provide detailed proofs of recoverability results for each distribution in m-DAG C2. These use the fact that the following conditional independence properties hold in this m-DAG:

$$M_X \perp (X, Z_2) \mid Y, Z_1$$
$$M_{Z_2} \perp (X, Z_2) \mid Y, Z_1$$
$$M_Y \perp (X, Z_2, Y) \mid Z_1$$

- Recoverability of joint distribution

$$P(Y^{\boldsymbol{M=0}}, X^{\boldsymbol{M=0}}, \boldsymbol{Z_2^{M=0}}, \boldsymbol{Z_1}) = P(Y^{\boldsymbol{M=0}}, X^{\boldsymbol{M=0}}, \boldsymbol{Z_2^{M=0}}|\boldsymbol{Z_1}) \times P(\boldsymbol{Z_1})$$

(by Bayes rule)

$$= P(Y^{\boldsymbol{M=0}}, X^{\boldsymbol{M=0}}, \boldsymbol{Z_2^{M=0}}|\boldsymbol{Z_1}, M_Y = 0) \times P(\boldsymbol{Z_1})$$

(by conditional independence properties)

$$= P(Y|\boldsymbol{Z_1}, M_Y = 0) \times P(X^{\boldsymbol{M=0}}, \boldsymbol{Z_2^{M=0}}|\boldsymbol{Z_1}, Y, M_Y = 0) \times P(\boldsymbol{Z_1})$$

(by consistency & Bayes rule)

$$= P(Y|\boldsymbol{Z_1}, M_Y = 0) \times P(X^{\boldsymbol{M=0}}, \boldsymbol{Z_2^{M=0}}|\boldsymbol{Z_1}, Y, \boldsymbol{M} = \boldsymbol{0}) \times P(\boldsymbol{Z_1})$$

(by conditional independence properties)

$$= P(Y|\boldsymbol{Z_1}, M_Y = 0) \times P(X, \boldsymbol{Z_2}|\boldsymbol{Z_1}, Y, \boldsymbol{M} = \boldsymbol{0}) \times P(\boldsymbol{Z_1})$$

(by consistency)

- Recoverability of marginal distribution of X

$$P(X^{\boldsymbol{M=0}}) = \sum_{\boldsymbol{Z_1}, Y} P(X^{\boldsymbol{M=0}}|\boldsymbol{Z_1}, Y^{\boldsymbol{M=0}}) \times P(Y^{\boldsymbol{M=0}}|\boldsymbol{Z_1}) \times P(\boldsymbol{Z_1})$$

(by Bayes rule)

$$= \sum_{\boldsymbol{Z_1}, Y} P(X^{\boldsymbol{M=0}}|\boldsymbol{Z_1}, Y^{\boldsymbol{M=0}}, M_X = 0, M_Y = 0) \times P(Y^{\boldsymbol{M=0}}|\boldsymbol{Z_1}, M_Y = 0) \times P(\boldsymbol{Z_1})$$

(by conditional independence properties)

$$= \sum_{\boldsymbol{Z_1}, Y} P(X|\boldsymbol{Z_1}, Y, M_X = 0, M_Y = 0) \times P(Y|\boldsymbol{Z_1}, M_Y = 0) \times P(\boldsymbol{Z_1})$$

(by consistency)

- Recoverability of marginal distribution of Y

$P(Y^{\boldsymbol{M=0}}) = \sum_{\boldsymbol{Z_1}} P(Y^{\boldsymbol{M=0}}|\boldsymbol{Z_1}) \times P(\boldsymbol{Z_1})$

$= \sum_{\boldsymbol{Z_1}} P(Y^{\boldsymbol{M=0}}|\boldsymbol{Z_1}, M_Y = 0) \times P(\boldsymbol{Z_1})$ (by conditional independence properties)

$= \sum_{\boldsymbol{Z_1}} P(Y|\boldsymbol{Z_1}, M_Y = 0) \times P(\boldsymbol{Z_1})$ (by consistency)

- Recoverability of conditional distribution of Y

$$P\left(Y^{\boldsymbol{M=0}}|X^{\boldsymbol{M=0}}, \boldsymbol{Z_2^{M=0}}, \boldsymbol{Z_1}\right) = \frac{P\left(Y^{\boldsymbol{M=0}}, X^{\boldsymbol{M=0}}, \boldsymbol{Z_2^{M=0}}|\boldsymbol{Z_1}\right)}{P(X^{\boldsymbol{M=0}}, \boldsymbol{Z_2^{M=0}}|\boldsymbol{Z_1})}$$

$$= \frac{P(Y|\boldsymbol{Z_1}, M_Y=0) \times P(X, \boldsymbol{Z_2}|\boldsymbol{Z_1}, Y, \boldsymbol{M=0})}{\sum_Y P(Y|\boldsymbol{Z_1}, M_Y=0) \times P(X, \boldsymbol{Z_2}|\boldsymbol{Z_1}, Y, \boldsymbol{M=0})}$$ (per proof for joint distribution above)

Recoverability results for m-DAG G2

Next, we provide detailed proofs of recoverability results for each distribution in m-DAG G2. These use the fact that the following conditional independence properties hold in this m-DAG:

$M_X \perp (X, Z_2, Y) \mid Z_1$
$M_{Z_2} \perp (X, Z_2, Y) \mid Z_1$
$M_Y \perp (X, Z_2) \mid Z_1, Y$

- Non-recoverability of joint distribution

This result follows from Theorem 3 of Mohan and Pearl,[6] which states that a necessary condition for the recoverability of the joint distribution is that none of the variables be a cause of their own missingness indicator. In m-DAG G2, Y is a cause of M_Y and thus this condition is violated.

- Recoverability of marginal distribution of X

$P(X^{\boldsymbol{M=0}}) = \sum_{\boldsymbol{Z_1}} P(X^{\boldsymbol{M=0}}|\boldsymbol{Z_1}) \times P(\boldsymbol{Z_1})$

$= \sum_{\boldsymbol{Z_1}} P(X^{\boldsymbol{M=0}}|\boldsymbol{Z_1}, M_X = 0) \times P(\boldsymbol{Z_1})$ (by conditional independence properties)

$= \sum_{\boldsymbol{Z_1}} P(X|\boldsymbol{Z_1}, M_X = 0) \times P(\boldsymbol{Z_1})$ (by consistency)

- Non-recoverability of marginal distribution of Y

This result also follows from Theorem 3 of Mohan and Pearl [6], as Y is a cause of M_Y.

- Non-recoverability of conditional distribution of Y

This result follows from Corollary 2 of Mohan and Pearl,[6] which states the conditional distribution of an outcome variable given other variables is not recoverable if the outcome variable is a cause of its own missingness indicator, which is the case for Y in m-DAG G2.

REFERENCES FOR APPENDIX S1-S3

1. Moreno-Betancur M, Lee KJ, Leacy FP, White IR, Simpson JA, Carlin JB. Canonical Causal Diagrams to Guide the Treatment of Missing Data in Epidemiologic Studies. *Am J Epidemiol*. 2018;187(12):2705-2715.
2. Mohan K. Graphical Models for Inference with Missing Data. Dissertation. 2017. https://escholarship.org/uc/item/6mk2b174.
3. Nabi R, Bhattacharya R. On Testability and Goodness of Fit Tests in Missing Data Models. In: *Proceedings of the 39th Conference on Uncertainty in Artificial Intelligence, PMLR*. ; 2023:216:1467–147.
4. Guo A, Zhao J, Nabi R. Sufficient identification conditions and semiparametric estimation under missing not at random mechanisms. In: *Proceedings of the 39th Conference on Uncertainty in Artificial Intelligence, PMLR*. ; 2023:216:777-787.
5. Zhang J, Dashti SG, Carlin JB, Lee KJ, Moreno-Betancur M. Recoverability and estimation of causal effects under typical multivariable missingness mechanisms. *Biometrical J*. 2024;66(2200326).
6. Mohan K, Pearl J. Graphical Models for Recovering Probabilistic and Causal Queries from Missing Data. In: Ghahramani Z, Welling M, Cortes C, Lawrence N, Weinberger K, eds. *Advances in Neural Information Processing Systems 27 (NIPS 2014)*. Red Hook, NY: Curran Associates, Inc; 2014:1520-1528.

FIGURES AND TABLES FOR APPENDIX S1-S3

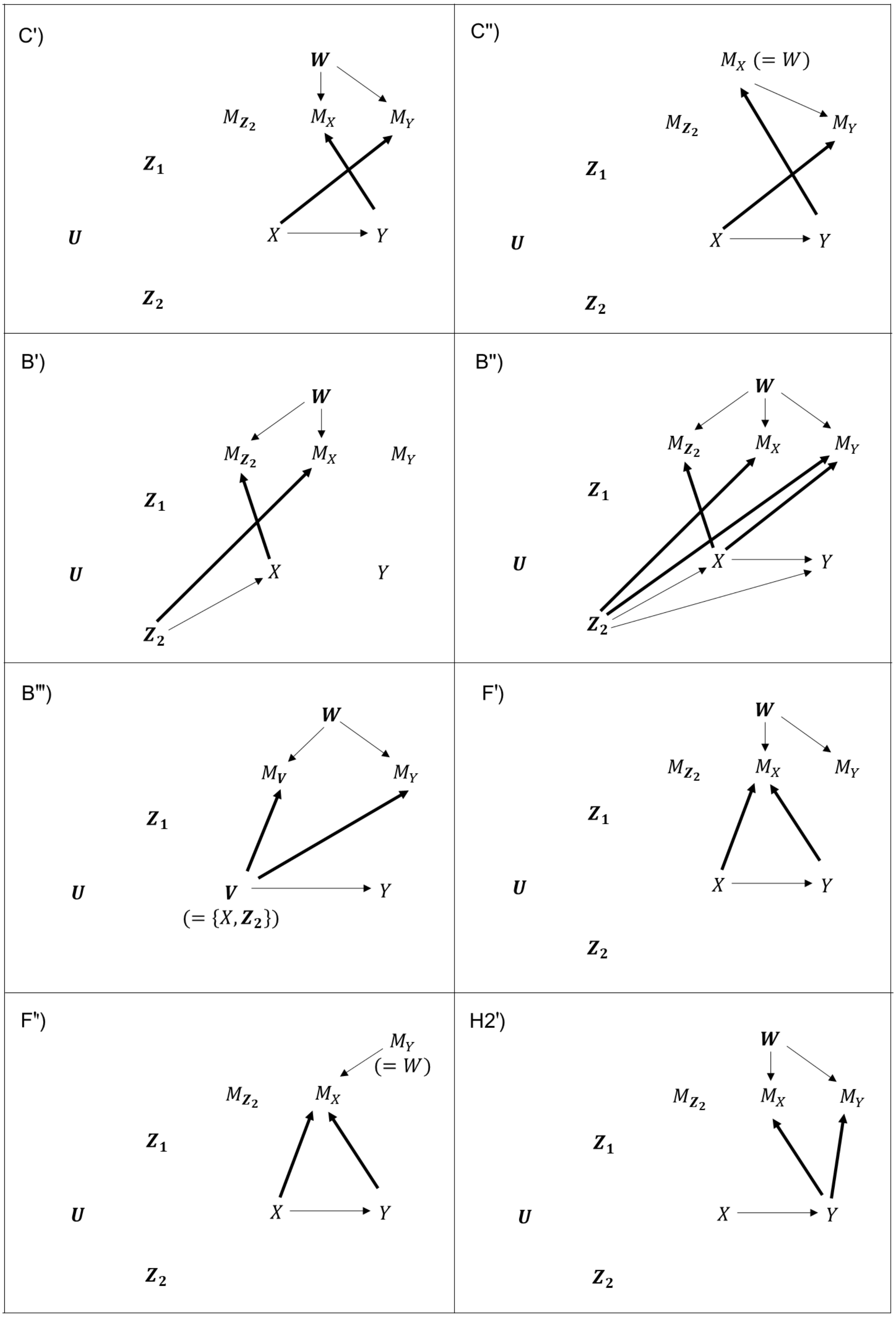

Figure S1. Reduced versions of canonical m-DAGs C, B, F and H2 used in the proofs in Appendix S1-S2

Table S1. Two models with distributions compatible with m-DAG F' in Figure S1, used in a proof in Appendix S2 [a]

x	y	m_X	m_Y	$P^{\mathcal{M}_1}(x, y, m_X, m_Y)$	$P^{\mathcal{M}_2}(x, y, m_X, m_Y)$
0	0	0	0	0	0
0	1	0	0	1/32	1/32
1	0	0	0	3/32	3/32
1	1	0	0	2/16	2/16
0	0	1	0	1/16	1/16
0	1	1	0	1/16	0
1	0	1	0	1/16	1/16
1	1	1	0	1/16	2/16
0	0	0	1	0	0
0	1	0	1	1/32	1/32
1	0	0	1	3/32	3/32
1	1	0	1	2/16	2/16
0	0	1	1	1/16	1/16
0	1	1	1	1/16	0
1	0	1	1	1/16	1/16
1	1	1	1	1/16	2/16

[a] Measured variables other than $X, Y, M_X,\ M_Y$ are assumed to follow the same distribution in both models and be independent of all other variables per m-DAG F'

Table S2. Observed distribution with missing data common to models $\mathcal{M}_1$and $\mathcal{M}_2$, used in a proof in Appendix S2 [a]

x^*	y^*	m_X	m_Y	$P(x^*, y^*, m_X, m_Y)$
0	0	0	0	0
0	1	0	0	1/32
1	0	0	0	3/32
1	1	0	0	2/16
0	Missing	0	1	1/32
1	Missing	0	1	7/32
Missing	0	1	0	2/16
Missing	1	1	0	2/16
Missing	Missing	1	1	4/16

[a] Observed distribution of measured variables other than $X, Y, M_X,\ M_Y$ is identical for models $\mathcal{M}_1$ and $\mathcal{M}_2$ by construction
